# DNA methylation across the genome in aged human skeletal muscle tissue and muscle-derived cells: the role of HOX genes and physical activity

**DOI:** 10.1038/s41598-020-72730-z

**Published:** 2020-09-21

**Authors:** D. C. Turner, P. P. Gorski, M. F. Maasar, R. A. Seaborne, P. Baumert, A. D. Brown, M. O. Kitchen, R. M. Erskine, I. Dos-Remedios, S. Voisin, N. Eynon, R. I. Sultanov, O. V. Borisov, A. K. Larin, E. A. Semenova, D. V. Popov, E. V. Generozov, C. E. Stewart, B. Drust, D. J. Owens, I. I. Ahmetov, A. P. Sharples

**Affiliations:** 1 Institute for Physical Performance, Norwegian School of Sport Sciences (NiH), Oslo, Norway; 2 Stem Cells, Ageing and Molecular Physiology Unit, Exercise Metabolism and Adaptation Research Group, Research Institute for Sport and Exercise Sciences, Liverpool John Moores University, Liverpool, UK; 3 Institute for Science and Technology in Medicine (ISTM), School of Pharmacy & Bioengineering, Keele University, Staffordshire, UK; 4 Exercise Metabolism and Adaptation Research Group, Research Institute for Sport and Exercise Sciences, Liverpool John Moores University, Liverpool, UK; 5 Exercise Biology Group, Faculty of Sport and Health Sciences, Technical University of Munich, Munich, Germany; 6 Centre for Genomics and Child Health, Blizard Institute, Barts and the London School of Medicine and Dentistry, Queen Mary University of London, London, UK; 7 Institute of Sport, Exercise and Health, University College London, London, UK; 8 Orthopedics Department, University Hospitals of the North Midlands, Keele University, Staffordshire, UK; 9 Institute for Health and Sport (iHeS), Victoria University, Footscray, VIC Australia; 10 Department of Molecular Biology and Genetics, Federal Research and Clinical Center of Physical-Chemical Medicine of Federal Medical Biological Agency, Moscow, Russia; 11 Institute for Genomic Statistics and Bioinformatics, University Hospital Bonn, Bonn, Germany; 12 Laboratory of Exercise Physiology, Institute of Biomedical Problems of the Russian Academy of Sciences, Moscow, Russia; 13 Laboratory of Molecular Genetics, Kazan State Medical University, Kazan, Russia; 14 School of Sport, Exercise and Rehabilitation Sciences, College of Life and Environmental Sciences, University of Birmingham, Birmingham, UK; 15 Department of Physical Education, Plekhanov Russian University of Economics, Moscow, Russia

**Keywords:** Epigenomics, Physiology, Ageing, Epigenetics, DNA methylation, Stem cells, Adult stem cells, Ageing, Epigenetic memory, Stem-cell differentiation

## Abstract

Skeletal muscle tissue demonstrates global hypermethylation with age. However, methylome changes across the time-course of differentiation in aged human muscle derived cells, and larger coverage arrays in aged muscle tissue have not been undertaken. Using 850K DNA methylation arrays we compared the methylomes of young (27 ± 4.4 years) and aged (83 ± 4 years) human skeletal muscle and that of young/aged heterogenous muscle-derived human primary cells (HDMCs) over several time points of differentiation (0, 72 h, 7, 10 days). Aged muscle tissue was hypermethylated compared with young tissue, enriched for; pathways-in-cancer (including; focal adhesion, MAPK signaling, PI3K-Akt-mTOR signaling, p53 signaling, Jak-STAT signaling, TGF-beta and notch signaling), rap1-signaling, axon-guidance and hippo-signalling. Aged cells also demonstrated a hypermethylated profile in pathways; axon-guidance, adherens-junction and calcium-signaling, particularly at later timepoints of myotube formation, corresponding with reduced morphological differentiation and reductions in MyoD/Myogenin gene expression compared with young cells. While young cells showed little alterations in DNA methylation during differentiation, aged cells demonstrated extensive and significantly altered DNA methylation, particularly at 7 days of differentiation and most notably in focal adhesion and PI3K-AKT signalling pathways. While the methylomes were vastly different between muscle tissue and HDMCs, we identified a small number of CpG sites showing a hypermethylated state with age, in both muscle tissue and cells on genes *KIF15*, *DYRK2*, *FHL2*, *MRPS33*, *ABCA17P*. Most notably, differential methylation analysis of chromosomal regions identified three locations containing enrichment of 6–8 CpGs in the HOX family of genes altered with age. With *HOXD10*, *HOXD9*, *HOXD8*, *HOXA3*, *HOXC9*, *HOXB1*, *HOXB3*, *HOXC-AS2* and *HOXC10* all hypermethylated in aged tissue. In aged cells the same HOX genes (and additionally *HOXC-AS3*) displayed the most variable methylation at 7 days of differentiation versus young cells, with *HOXD8*, *HOXC9*, *HOXB1* and *HOXC-AS3* hypermethylated and *HOXC10* and *HOXC-AS2* hypomethylated. We also determined that there was an inverse relationship between DNA methylation and gene expression for *HOXB1*, *HOXA3* and *HOXC-AS3*. Finally, increased physical activity in young adults was associated with oppositely regulating *HOXB1* and *HOXA3* methylation compared with age. Overall, we demonstrate that a considerable number of HOX genes are differentially epigenetically regulated in aged human skeletal muscle and HDMCs and increased physical activity may help prevent age-related epigenetic changes in these HOX genes.

## Introduction

Maintaining skeletal muscle mass and function into older age is fundamental for human health-span and quality of life^1^. Five to ten percent of older adults have sarcopenia^2,3^, which is characterized by reductions in muscle mass and strength^4^. This loss of muscle mass and strength leads to frailty, increased incidence of falls, hospitalization and morbidity^4–11^. Annual costs of fragility are estimated to be 39/32 billion (Euros/USD) for European and USA fragility fractures respectively, with the cost of sarcopenia estimated to be £2 billion in the UK^12^. With an increasing aged population, these costs are likely to increase with time.

A primary hallmark of ageing is the alteration of the epigenetic landscape. Epigenetics encompasses the interaction between lifestyle/environmental factors and modifications to DNA and histones, without changes to the inherited DNA sequence^13,14^. DNA methylation is the most studied epigenetic modification and involves the addition of a covalent methyl group to the 5′ position of the pyrimidine ring of a cytosine (5mC). Increased methylation (hypermethylation) to cytosine–guanine (C–G) pairings (CpG sites), especially in CpG-rich regions such as gene promoters, typically leads to reduced capacity for the transcriptional apparatus to bind to these regions, suppressing gene expression^14^. Methylated CpG islands in promoters also leads to a tight compaction of adjacent chromatin via the recruitment of chromatin modifying protein/protein-complexes, further silencing gene expression. In contrast, reduced methylation (hypomethylation) provides a more favorable DNA landscape for the transcriptional apparatus to bind to these regions, as well as more relaxed chromatin, enabling gene expression to occur.

DNA methylation in aged skeletal muscle occurs at tissue-specific genes^15^. However, aged muscle also has the smallest overlap with other aged tissue types, suggesting skeletal muscle is unique in comparison with other tissues in its epigenetic aging processes^15^. Indeed, it has recently been demonstrated that the methylation status of approximately 200 CpG sites can accurately predict chronological age in skeletal muscle tissue^16^. But that this muscle ‘clock’ only shares 16 of its CpG’s with the original 353 CpG pan-tissue Horvath epigenetic clock^16,17^. Further, using DNA methylation arrays with coverage of ~ 450,000 CpG sites^18^, Zykovich et al*.* demonstrated that compared with young human skeletal muscle, aged skeletal muscle is hypermethylated across the genome. Moreover, our group has demonstrated that mouse skeletal muscle cells exposed to a high dose of inflammatory stress in early proliferative life retained hypermethylation of *MyoD* (a muscle-specific regulatory factor) 30 population doublings later^19^. This suggested that inflamed proliferative aging in muscle cells leads to a retained accumulation of DNA methylation. Finally, lifelong physical activity^20^, endurance and resistance exercise have been associated with evoking hypomethylation of the genome in young skeletal muscle^21,22^. This contrasts with the hypermethylation observed with aging, suggesting that exercise or increased physical activity may reverse some age-related changes in DNA methylation.

Skeletal muscle fibers are post-mitotic as they contain terminally differentiated/fused nuclei (myonuclei); thus, repair and regeneration of skeletal muscle tissue is mediated by a separate population of resident stem cells (satellite cells) that can divide. Once activated, satellite cells proliferate and migrate to the site of injury to differentiate and fuse with the existing fibers to enable repair. Targeted gene analysis demonstrated altered DNA methylation during differentiation of muscle cells into myotubes in-vitro^23^. This included altered methylation of MyoD^24^, Myogenin^25^ and Six1^26^. While skeletal muscle cells derived from aged individuals display similar proliferative capacity and time to senescence as young adult cells^27,28^, they have been shown to display impaired differentiation and fusion into myotubes^29–45^. However, a small number of studies have not found an effect of age on the differentiation capacity of isolated cells^27,46,47^. A single study assessed DNA methylation across the genome (450 K CpG sites) in aged versus young adult muscle cells^27^ and showed genome-wide hypermethylation in aged cells as well as aged tissue^27^.

To date, there has been no report of genome-wide DNA methylation dynamics during the entire time-course of muscle cell differentiation, or how age modulates these dynamics. Furthermore, the latest, larger coverage methylation arrays have not yet been implemented in aged muscle tissue. Therefore, the objectives of the current study were: (1) To describe the dynamics of the human DNA methylome in aged and young adult skeletal muscle tissue and heterogenous muscle-derived human primary cells (HDMCs) over an extensive time-course of differentiation; 0 h (30 min post transfer to differentiation media), 72 h (hours), 7 d (days) and 10 d using high coverage 850 K CpG arrays. (2) To identify if methylation patterns are similar or different in HDMCs compared to skeletal muscle tissue. (3) To test whether increasing physical activity levels is associated with counteracting the changes in DNA methylation observed in aging.

## Methods

### Skeletal muscle biopsies and primary cell isolations

For young adults (n = 9, male, 27 ± 4.4 years-old), skeletal muscle tissue (~ 150 mg) was obtained from the vastus lateralis via a conchotome biopsy. Consent and ethical approval were granted for the collection of muscle tissue under NREC, UK approval (16/WM/010) or LJMU, UK local ethics committee approvals [H19/SPS/028 & H15/SPS/031)**.** Six (out of 9) of the young adult’s tissue (male, 28 ± 5.3 years) baseline (at rest) array data was derived from Seaborne et al*.* (2018). This is because we used this baseline tissue to derive cells (detailed below) for DNA methylation analysis of differentiating human muscle derived cells in the present study. Schematic of study design can be seen in Suppl. Figure 1. For older adults (n = 5, 2 men/3 women, 83 ± 4 years), tissue biopsies were obtained during elective orthopedic surgeries from University Hospitals of the North Midlands, from the vastus lateralis (knee surgery, n = 2) or gluteus medius muscles (hip surgery, n = 3), under consent and ethical NREC, UK approval 18/WM/0187. DNA and RNA were isolated from these young and aged tissue samples. DNA samples from all 9 young and 5 aged adults were analysed for DNA methylation arrays (detailed below), and a subset were analysed for gene expression (young n = 4, aged n = 5). Heterogenous muscle-derived human primary cells (HDMCs) from a subset of young adult and aged tissue samples were isolated as per our previous work^21,22,48–51^. Briefly, approximately 100 mg biopsy tissue was immediately (~ 10–30 min) transferred to a sterile class II biological safety cabinet in pre-cooled (4 °C) transfer media (Hams F-10, 2% hi-FBS, 100 U/ml penicillin, 100 μg/ml streptomycin and 2.5 µg/ml amphotericin-B). Any visible connective and adipose tissue were removed using sterile scalpels and muscle tissue was thoroughly washed 2× in sterile PBS (containing 100 U/ml penicillin, 100 µg/ml streptomycin, 2.5 µg/ml amphotericin-B). PBS was removed and the muscle tissue was minced in the presence of 0.05% Trypsin/0.02% EDTA and all contents (tissue and trypsin) were transferred to a magnetic stirring platform at 37 °C for 10 min. The addition of 0.05% Trypsin/0.02% EDTA and titration was repeated on any remaining tissue. The supernatant was collected from both procedures and horse serum (HS) was added at 10% of the total supernatant volume to neutralize the trypsin. The supernatant was centrifuged at 340 g for 5 min where the supernatant and cell pellet were both plated in separate pre-gelatinised (0.2% gelatin) T25 flasks containing 7.5 ml fresh pre-heated growth media/GM (GM; Ham’s F10 nutrient mix supplemented with 10% hi-NCS, 10% hi-FBS, 100 U/ml penicillin, 100 µg/ml streptomycin, 2.5 µg/ml amphotericin B and 5 mM L-glutamine). Once confluent, cells were trypsinised and reseeded into larger T75′s to expand the cell population. HDMCs were seeded onto gelatin coated 6 well plates, at a density of 9 × 10^5^ cells/ml in 2 ml of GM until ~ 90% confluency was attained (taking ~ 48 h). GM was removed, and cells underwent 3× PBS washes before switching to low serum differentiation media (DM; Ham’s F10 nutrient mix supplemented with 2% hiFBS, 100 U/ml penicillin, 100 µg/ml streptomycin, 2.5 µg/ml amphotericin B and 5 mM L-glutamine). HMDCs were differentiated for a total of 10 days (d) which received a 1 ml top up of DM at 72 h and 7 d timepoints. Cells were lysed for DNA and RNA at 0 h (30 min in DM), 72 h, 7 d and 10 d, at the same time of day (approx. 08.00–11.00) to minimize the impact of circadian oscillation^52^. All experiments were carried out below passage 10 to prevent senescence. We undertook methylation arrays on DNA isolated from: 0 h young (n = 7), 0 h aged (n = 4), 72 h young (n = 7), 72 h aged (n = 4), 7 d young (n = 6), 7d aged (n = 3), 10 d young (n = 2) and 10 d aged (n = 4). Gene expression was analysed using young (n = 4, 7 d) and aged (n = 3, 7 d) cells. It’s worth noting that we had n = 3–4 for most conditions, however in the 10 d young cells condition, some DNA did not pass QA/QC for the arrays, and we had no cells left for these participants. Therefore, unfortunately we could only run n = 2 for this single condition. Therefore, results for this condition should be viewed with this caveat in mind. A schematic of the overarching experimental design can be found in Suppl. Figure 1.

### Myogenicity and morphology measurements

Following isolations, attached single cells were fixed prior to staining and fluorescent immunocytochemistry analysis to determine myogenicity (via desmin positivity) of the isolated young and aged muscle derived cultures. Briefly, 2 × 10^4^ cells were seeded onto 3× wells of a 6-well plate and were incubated for 24 h in GM. Existing media was removed and cells were washed 3 × in PBS before fixation using the graded methanol/acetone method (50:25:25 TBS:methanol:acetone for 15 min followed by 50:50 methanol:acetone for 10-15 min) after which cells were permeabilised in 0.2% Triton X-100 and blocked using 5% goat serum (Sigma-Aldrich, UK) in TBS for 30 min. Cells were washed 3 × in TBS and incubated overnight (4 °C) in 300 μl of TBS (with 2% goat serum and 0.2% Triton X-100) containing primary anti-desmin antibody (1:50; ab15200, Abcam, UK). After overnight incubation, cells were washed 3× in TBS and incubated at RT for 3 h in 300 μl of secondary antibody solution (TBS, 2% goat serum and 0.2% Triton X-100) containing the secondary antibody, anti-rabbit TRITC (1:75; T6778, Sigma-Aldrich, UK) to counterstain desmin. Finally, cells were washed again 3× in TBS, prior to counterstaining nuclei using 300 μl of DAPI solution at a concentration of (300 nM; D1306, Thermo Fisher Scientific, UK) for 30 min. Immunostained cells were then visualised using a fluorescent microscope (Nikon, Eclipse Ti-S, Japan or Olympus IX83, Japan) and imaged using corresponding software (Nikon, NIS Elements and Olympus FV10-ASW 4.2). Myoblasts, myotubes and nuclei were visualized using TRITC (Desmin, Excitation: 557 nm, Emission: 576 nm) and DAPI (Excitation: 358 nm, Emission: 461 nm) filter cubes. All immunostained samples were imported to Fiji/ImageJ (version 2.0.0) software for subsequent calculations. There was no difference (p = 0.86) in myogenic cell proportions in aged (35 ± 5%) versus young cells (34 ± 9%). Therefore, the aged and young muscle derived cells were matched for their desmin positivity (% myoblasts) prior to differentiation experiments and downstream DNA methylation and gene expression analysis. To determine myotube differentiation and formation at each time point, cells were imaged using light microscopy (Olympus, CKX31, Japan) at 0, 72 h, 7 and 10 d of differentiation.

### DNA isolation and bisulfite conversion

Prior to DNA isolation, tissue samples were homogenized for 45 s at 6,000 rpm × 3 (5 min on ice in between intervals) in lysis buffer (180 µl buffer ATL with 20 µl proteinase K) provided in the DNeasy spin column kit (Qiagen, UK) using a Roche Magnalyser instrument and homogenization tubes containing ceramic beads (Roche, UK). DNA was then isolated using the DNAeasy kit (Qiagen, UK) according to manufacturer’s instructions. Cells were lysed in 180 µl PBS containing 20 µl proteinase K, scraped from wells, and then isolated using DNAeasy kits (Qiagen, UK) using the same method as described above with the tissue lysates. The DNA was then bisulfite converted using the EZ DNA Methylation Kit (Zymo Research, CA, United States) as per the manufacturers instructions.

### Infinium Methylation EPIC Beadchip array

All DNA methylation experiments were performed in accordance with Illumina manufacturer instructions for the Infinium Methylation EPIC 850K BeadChip Array (Illumina, USA). Methods for the amplification, fragmentation, precipitation and resuspension of amplified DNA, hybridisation to EPIC beadchip, extension and staining of the bisulfite converted DNA (BCD) can be found in detail in our open access methods paper^49^. EPIC BeadChips were imaged using the Illumina iScan System (Illumina, United States).

### DNA methylation analysis, CpG enrichment analysis (GO and KEGG pathways), differentially modified region (DMR) analysis and Self Organising Map (SOM) profiling

Following DNA methylation quantification via MethylationEPIC BeadChip array, raw .IDAT files were processed using Partek Genomics Suite V.7 (Partek Inc. Missouri, USA) and annotated using the MethylationEPIC_v-1-0_B4 manifest file. We first checked the average detection p-values. The highest average detection p-value for the samples was 0.0023 (Suppl. Figure 2a), which is well below the recommended 0.01 in the Oshlack workflow^53^. We also produced density plots of the raw intensities/signals of the probes (Suppl. Figure 2b). These demonstrated that all methylated and unmethylated signals were over 11.5 (mean was 11.52 and median was 11.8), and the difference between median methylation and median unmethylated signal was 0.56^53^. Upon import of the data into Partek Genomics Suite we removed probes that spanned X and Y chromosomes from the analysis due to having both males and females in the study design, and although the average detection p-value for each samples was on average very low (no higher than 0.0023) we also excluded any individual probes with a detection p-value that was above 0.01 as recommended in^53^. Out of a total of 865,860 probes, removal of the X and Y chromosome probes and those with a detection p-value above 0.01 reduced the probe number to 846,233 (removed 19,627 probes). We also filtered out probes located in known single-nucleotide polymorphisms (SNPs) and any known cross-reactive probes using previously defined SNP and cross-reactive probe lists identified in earlier 850 K validation studies^54^. This resulted in a final list of 793,200 probes to be analysed. Following this, background normalisation was performed via functional normalisation (with noob background correction), as previously described^55^. Following functional normalisation, we also undertook quality control procedures of principle component analysis (PCA), density plots by lines as well as box and whisker plots of the normalised data for tissue samples (Suppl. Figures 2c,d,e respectively) and cell samples (Suppl. Figures 2f,g,h respectively). Any outlier samples were detected using PCA and the normal distribution of β-values. Outliers were then removed if they fell outside 2 standard deviations (SDs) (e.g. Suppl. Figure 2c) of the ellipsoids and/or if they demonstrated different distribution patterns to the samples of the same condition. Only one young adult male tissue sample was removed due to being outside 2 standard deviations outside samples from the same condition (Suppl. Figure 2c; sample with a strikethrough line). Following normalisation and quality control procedures, we undertook differential methylation analysis by converting β-values to M-values (M-value = log2( β/(1 − β)), as M-values show distributions that are more statistically valid for the differential analysis of methylation levels^56^. We undertook a 1-way ANOVA for comparisons of young and aged skeletal muscle tissue. For primary human muscle cells, we undertook a 2-way ANOVA for age (young vs. aged cells) x time (0, 72 h, 7 and 10 d) and also explored the ANOVA main effects for both age and time independently. We also performed planned contrasts within the differentiation time-course in both young adult cells alone and aged cells alone e.g. 0 h vs. 72 h, 0 h vs. 7 d 0 h vs. 10 d, as well as planned contrasts for young vs. aged cells at each time point of differentiation (0 h vs. 0 h, 72 h vs. 72 h, 7 d vs. 7 d, 10 d vs. 10 d in aged vs. young adult cells respectively). Any CpG with a False Discovery Rate (FDR) ≤ 0.05 was deemed significant. In some analyses, e.g. > 100,000 differentially methylated CpG sites were identified at FDR ≤ 0.05, so we also show results at a more stringent FDR of ≤ 0.01 or 0.001, or at FDR of ≤ 0.05 and ‘a change (difference in M-value) in methylation greater than 2′. These shorter lists of CpGs contain the most significant sites to enable sensible pathway analysis. We specify when a more stringent FDR than 0.05 was used in the results text. We then undertook CpG enrichment analysis on these differentially methylated CpG lists within gene ontology and KEGG pathways^57–59^ using Partek Genomics Suite and Partek Pathway. We also undertook differentially methylated region analysis (e.g. identifies where several CpGs are consistently differentially methylated within a short chromosomal location/region) using the Bioconductor package DMRcate (https://doi.org/10.18129/B9.bioc.DMRcate). Finally, in order to plot temporal changes in methylation across the time-course of muscle cell differentiation we undertook Self Organising Map (SOM) profiling of the change in mean methylation within each condition using Partek Genomics Suite.

### RNA isolation, primer design & gene expression analysis

Skeletal muscle tissue muscle was homogenised in tubes containing ceramic beads (﻿MagNA Lyser Green Beads, Roche, Germany) and 1 ml Tri-Reagent (Invitrogen, Loughborough, UK) for 45 s at 6,000 rpm × 3 (and placed on ice for 5 min at the end of each 45 s homogenization) using a Roche Magnalyser instrument (Roche, Germany). Human cells from differentiation experiments were also lysed in 300 µl Tri-Reagent for 5 min at RT and mechanically dissociated/lysed using a sterile scraper. RNA was then isolated as per Invitrogen’s manufacturer’s instructions for Tri-reagent. Then a one-step RT-PCR reaction (reverse transcription and PCR) was performed using QuantiFast SYBR Green RT-PCR one-step assay kits on a Rotorgene 3000Q. Each reaction was setup as follows; 4.75 μl experimental sample (7.36 ng/μl totalling 35 ng per reaction), 0.075 μl of both forward and reverse primer of the gene of interest (100 μM stock suspension), 0.1 μl of QuantiFast RT Mix (Qiagen, Manchester, UK) and 5 μl of QuantiFast SYBR Green RT-PCR Master Mix (Qiagen, Manchester, UK). Reverse transcription was initiated with a hold at 50 °C for 10 min (cDNA synthesis) and a 5-min hold at 95 °C (transcriptase inactivation and initial denaturation), before 40–50 PCR cycles of; 95 °C for 10 s (denaturation) followed by 60 °C for 30 s (annealing and extension). Primer sequences for genes of interest and reference genes are included in Table 1. All genes demonstrated no unintended targets via BLAST search and yielded a single peak after melt curve analysis conducted after the PCR step above. All relative gene expression was quantified using the comparative Ct (^∆∆^Ct) method^60^. For human cell differentiation analysis via measurement of myoD and myogenin, a pooled mean Ct for the 0 h young adult control samples were used as the calibrator when comparing aged vs. young adult cells. This approach demonstrated a low % variation in C_t_ value of 9.5 and 8.5% for myoD and myogenin, respectively. For HOX gene analysis between young and aged tissue and for the 7 d aged cells vs. 7 d young adult cells, the mean Ct of the young adult cells were used as the calibrator. The average, standard deviation and variations in Ct value for the B2M reference gene demonstrated low variation across all samples (mean ± SD, 13.12 ± 0.98, 7.45% variation) for the analysis of myoD and myogenin. For HOX gene analysis, the RPL13a reference gene variation was low in the human tissue (17.77 ± 1.71, 9.6% variation) and HDMCs (15.51 ± 0.59, 3.82% variation) experiments. The average PCR efficiencies of myoD and myogenin were comparable (94.69 ± 8.9%, 9.4% variation) with the reference gene B2M (89.45 ± 3.76%, 4.2% variation). The average PCR efficiencies of the all genes of interest in the tissue analysis of the HOX genes were also comparable (90.87 ± 3.17%, 3.39% variation) with the human reference gene RPL13a (92 ± 2.67%, 2.9% variation). Similarly, for the cell analysis, HOX genes of interest efficiencies were comparable (89.59 ± 4.41%, 4.93% variation 4.93%) with the reference gene RPL13a (89.57 ± 3.55%, 3.97% variation). Statistical analysis for HOX genes was performed using t-tests (aged tissue vs. young tissue and 7d aged versus 7d young).

**Table 1:** Primer sequences for targeted gene expression analysis.

Gene / Primer	Sequence	Product Length (Bp)	Transcript(s) determined
MyoD Forward	GCCACAACGGACGACTTCTA	117	NM_002478.5
MyoD Reverse	GTGCTCTTCGGGTTTCAGGA		
Myogenin Forward	TCCCAGATGAAACCATGCCC	103	NM_002479.6
Myogenin Reverse	AGGCCCCTGCTACAGAAGTA		
HOXC10 Forward	GCGAAAGAGGAGATAAAGGCAG	166	NM_017409.4
HOXC10 Reverse	TCTTGCTAATCTCCAGGCGG		
HOXB3 Forward	AACGCCTTACACTCCATGACC	70	Transcript variants 1–3 (NM_002146.4, NM_001330322.2, NM_001330323.1), Transcript variants X1-10 (XM_017024560.1, XM_011524708.3, XM_024450737.1, XM_011524720.3, XM_011524710.2, XM_006721854.3, XM_005257277.3, XM_011524719.2, XM_011524721.3, XM_011524726.2)
HOXB3 Reverse	TCTGGTGGGCTTTACCGAAG		
HOXC-AS2 Forward	GAACCTGGAGCCGGAAACTC	94	lncRNA (NR_047505.1)
HOXC-AS2 Reverse	CTGAGAGGGGCTGGTTAAGG		
HOXD8 Forward	GTGAAGGACGGGGAAACGAA	70	Transcript variants 1–3 (NM_019558.4, NM_001199746.2, NM_001199747.2)
HOXD8 Reverse	AATTTGTCAGGCCTTCGGCT		
HOXB1 Forward	ACTGCCGAAAGGTTGTAGGG	195	NM_002144.4
HOXB1 Forward	ATAGCTGTCAACCGCCTGAG		
HOXC9 Forward	GGACTCGCTCATCTCTCACG	115	NM_006897.3
HOXC9 Reverse	AAAATCGCTACAGTCCGGCA		
HOXA3 Forward	TCTACGGTGGCTACCCCTAC	173	Transcript variants 1–2 (NM_030661.4, NM_153631.2), Transcript variants X1-5 (XM_011515343.3, XM_006715715.2, XM_005249730.2, XM_005249732.4, XM_005249731.2)
HOXA3 Reverse	CTCACTCAGTTCGTGTGCCT		
HOXC-AS3 Forward	GTGGAGTAACAGCGCCATCT	117	lncRNA (NR_047506.1)
HOXC-AS3 Reverse	CGGGTTTTGTTGCGTCTTGT		
RPL13A Forward	GGCTAAACAGGTACTGCTGGG	105	NM_012423.4
RPL13A Reverse	AGGAAAGCCAGGTACTTCAACTT		
B2M Forward	GATGAGTATGCCTGCCGTGT	105	NM_004048.3
B2M Reverse	TGCGGCATCTTCAAACCTCC		

### Physical activity and DNA methylation

The human association study involved 30 physically active and endurance-oriented men of Eastern European descent (32.9 ± 9.9 years). The study was approved by the Ethics Committee of the Physiological Section of the Russian National Committee for Biological Ethics and Ethics Committee of the Federal Research and Clinical Center of Physical–chemical Medicine of the Federal Medical and Biological Agency of Russia. Written informed consent was obtained from each participant. The study complied with the guidelines set out in the Declaration of Helsinki. Physical activity was assessed using questionnaire. Participants were classified as mildly active (1–2 training sessions per week, n = 6), moderately active (3–4 training sessions per week, n = 8) and highly active (5–7 sessions per week, n = 16) participating in aerobic exercise for at least the last 6 months. Bisulfite conversion of genomic DNA was performed using the EpiMark Bisulfite Conversion Kit in accordance with the manufacturer's instructions. In the same analysis as described above for the aged muscle tissue and HDMCs data, methylome of the vastus lateralis in the physically active men was evaluated using the Infinium MethylationEPIC 850 K BeadChip Array (Illumina, USA) and imaged and scanned using the Illumina iScan System (Illumina, United States). Also as in the above analysis we filtered out probes located in known single-nucleotide polymorphisms (SNPs) and any known cross-reactive probes using previously defined SNP and cross-reactive probe lists^61^. Low quality probes were also filtered, as with the above analysis. The final analyses included 796,180 of 868,565 probes. Data were normalised using the same functional normalisation (with noob background correction), as in the aged tissue and HDMCs data, and as previously described^55^. Any outliers were interrogated via PCA, however all samples passed the QC and therefore there were no outliers. The methylation level of each CpG-site after normalization and filtering processes was represented as a β-value ranging from 0 (unmethylated) to 1 (fully methylated) in order to undertake multiple regression (as regression analysis performed better with finite values if values range from 0 to 1 with beta-values satisfying this criteria). Statistical analyses were conducted using PLINK v1.90, R (version 3.4.3) and GraphPad InStat (GraphPad Software, Inc., USA) software. Multiple regression was used for testing associations between the CpG-methylation level and physical activity adjusted for age and muscle fibre composition. With methylation level as the dependent variable, and physical activity and age as independent variables. P values < 0.05 were considered statistically significant to test the identified HOX CpG sites. The p value is given for the physical activity score adjusted for age and muscle fibre composition.

## Results

### Aged human skeletal muscle tissue is hypermethylated compared with young adult tissue

**Figure 1:**
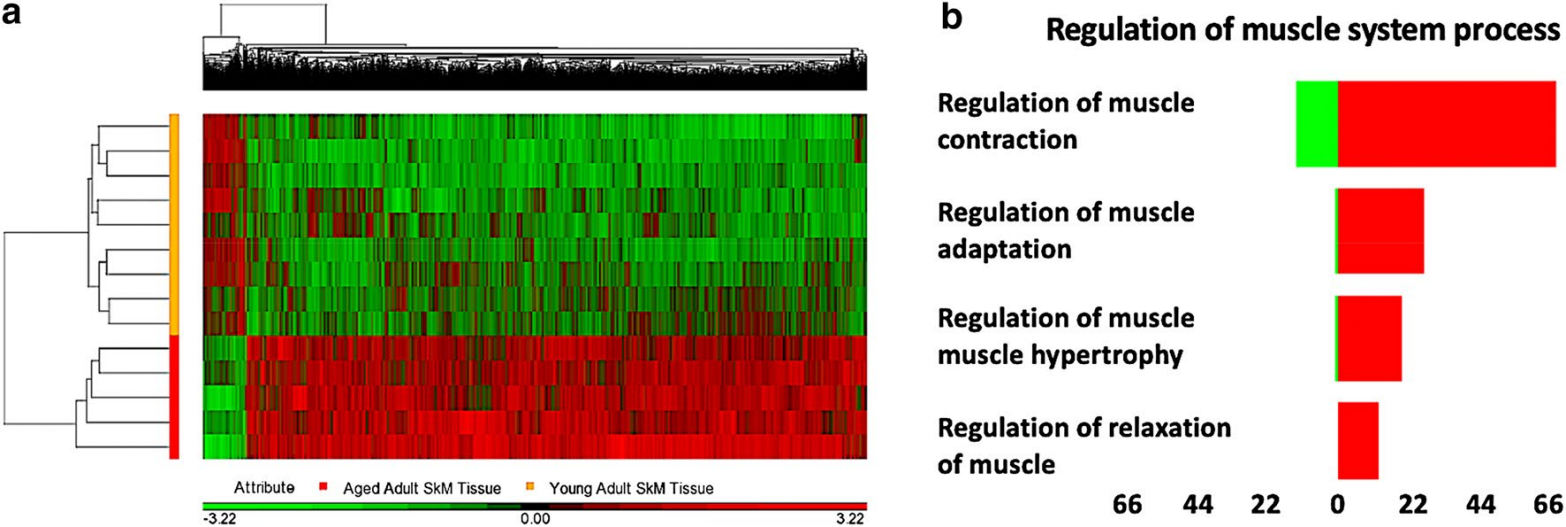
DNA methylation across the genome in aged human skeletal muscle tissue compared with young adult tissue. **(a)** Hierarchical clustering heatmap of the significantly differentially methylated CpG sites, depicting aged skeletal muscle containing a hypermethylated (RED) versus hypomethylated (GREEN) signature compared with young adult tissue. (**b)** CpG sites for most significantly enriched GO term containing the search term ‘muscle’; ‘regulation of muscle system process’ (RED hypermethylated and GREEN hypomethylated CpGs). X axis labels = number of DMPs.

There were 17,994 differentially methylated CpG positions (DMPs) between aged and young adult tissue at FDR ≤ 0.05 (Suppl. File 1a), and 6828 DMPs at a more stringent FDR of ≤ 0.01 (Suppl. File 1b). The overwhelming majority of the 6828 DMPs (93%) were hypermethylated in aged compared with young muscle (Fig. 1a**)**. Furthermore, DMPs were enriched in CpG islands (5018 out of 6828 DMPs) with the remaining in N-shores (354), S-Shelf (48), S-Shores (341), S-Shelf (69) and ‘other’ (998). Ninety nine percent of the DMPs (4976 out of 5018) located in CpG islands were also hypermethylated with age compared to young adult tissue (Suppl. File 1c). After gene ontology analysis on these DMPs (Suppl. File 1d), hypermethylation was enriched within the three overarching gene ontology terms: ‘Biological process’ (Suppl. Figure 3a), ‘cellular component’ (Suppl. Figure 3b), and ‘molecular function’ (Suppl. Figure 3c). Within the GO terms that included the search term ‘muscle’, the most significantly enriched was ‘regulation of muscle system process’ (Fig. 1b, DMP list Suppl. File 1e). Within this GO term was ‘regulation of muscle contraction’ (CpG list Suppl. file 1f). Further, we found hypermethylation enrichment in KEGG pathways: ‘Pathways in cancer’, ‘Rap1 signaling’, ‘Axon guidance’ and ‘Hippo signaling’ (Suppl. File 1g). Within the top enriched pathway, ‘pathways in cancer’, 96% (266 out of 277 DMPs) were hypermethylated and only 4% (11 DMPs) were hypomethylated in aged compared with young adult muscle tissue (Suppl. Figure 4; Suppl. File 1h). Differentially methylated regions (DMRs) were also analysed between young and aged skeletal muscle tissue (Suppl. File 1i). The top 5 DMRs were identified as: chr8:22,422,678–22,423,092 (415 bp) within a CpG island of the SORBS3 gene with 6 CpG’s that were all hypermethylated in aged versus young adult tissue. Also, chr6:30,653,732–30,655,720 (1989 bp) within a CpG island of the PPPR1 gene contained 8 CpG’s that were all hypermethylated compared with young adult muscle. Chr20:13,200,939–13,202,437 (1499 bp) within a CpG island of the promoter of gene ISM, contained 8 CpG sites were also all hypermethylated. Similarly, the gene PDE4D1P on Chr1:144,931,376–144,932,480 (1105 bp) contained 6 CpG sites that spanned its promoter within a CpG island, once again demonstrating hypermethylation. The only gene demonstrating the opposite direction in methylation status (hypomethylation) in aged tissue was the gene C1orf132 (new gene name MIR29B2CHG), also on chromosome 1 (Chr1:207,990,896–207,991,936; 1041 bp), where 5 CpGs within this region were hypomethylated in opposition to the majority of gene regions that were hypermethylated in aged versus young tissue.

### Aged HDMCs displayed differential DNA methylation signatures versus young adults during the time-course of differentiation

**Figure 2:**
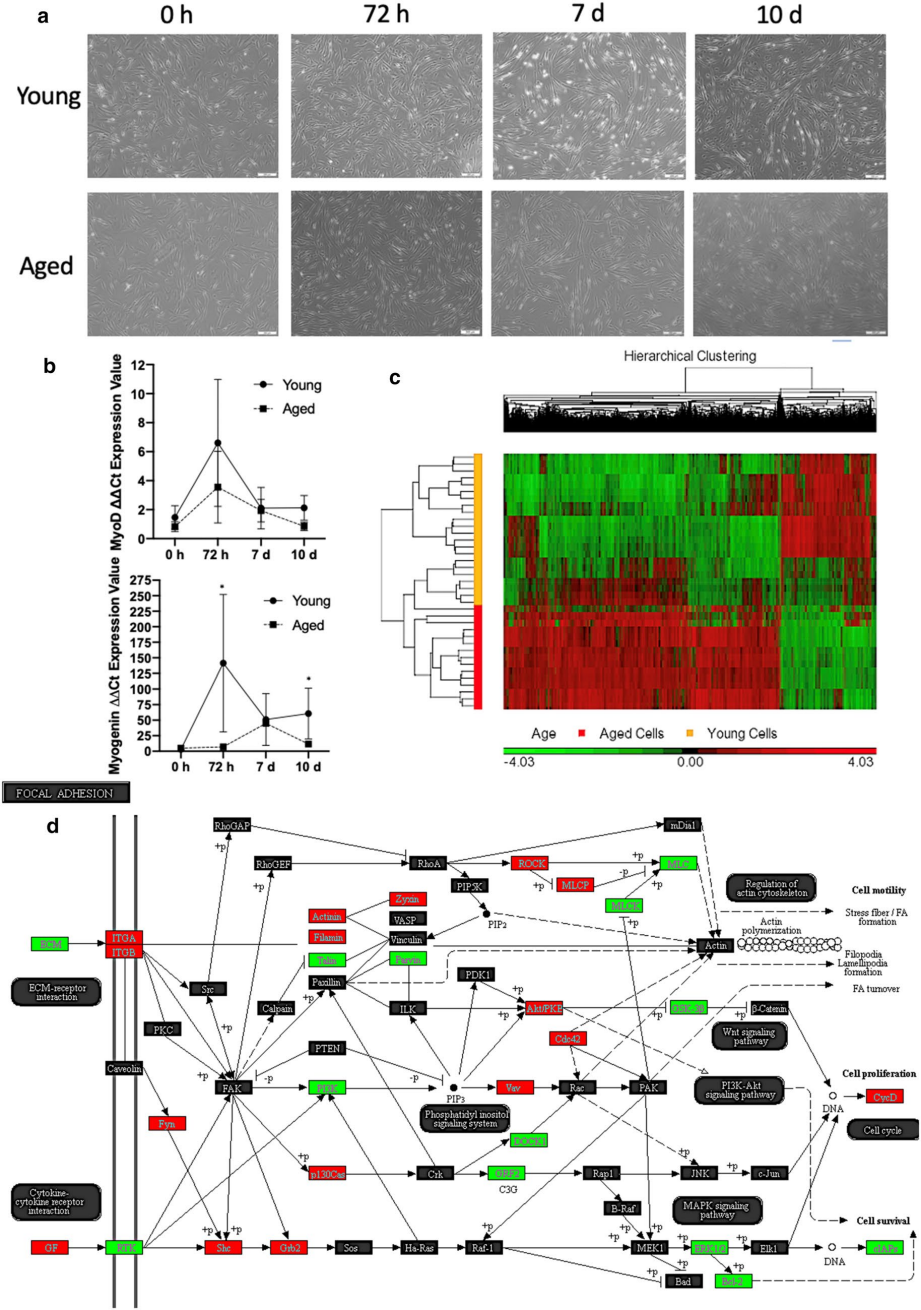
Aged and young adult HDMCs differentiated over 0, 72 h, 7d and 10 d and differences in DNA methylation. **(a)** Light microscope images of aged versus young HDMCs, depict fewer myotubes in aged versus young cells, particularly at 7 and 10 days. (**b)** MyoD and myogenin gene expression in aged versus young cells over the differentiation time course. Where, reductions in myogenin were observed at 72 h in aged cells as well as a delayed increase in the upregulation of myogenin compared with young cells. **(c)** Hierarchical clustering heatmap of significant DMPs between aged and young HDMCs across the entire time-course of differentiation (all time points of 0, 72 h, 7 d and 10 d). RED hypermethylated and GREEN hypomethylated. (**d)** Comparison of DNA methylation in aged versus young HDMCs at 7 d of differentiation (7 d aged vs. 7 d young adult cells) in most enriched KEGG pathway, ‘focal adhesion’. Copyright permission for KEGG pathway image obtained from Kanehisa Laboratories^57–59^. Note, the most significant (lowest p-value) DMP for each gene is used to colour this pathway image. Therefore, this is not always accurate where multiple DMPs occur for a single gene and the image is therefore only a visual representation of the overarching methylation profile in this pathway. Full and accurate DMP lists for 'focal adhesion' pathway in these conditions can be found in  Suppl. File 5i.

We analysed DNA methylation from differentiating HDMCs at 0 h (30 min post dropping to 2% serum), 72 h, 7 and 10 d. Both young and aged adult cells demonstrated morphological differentiation and myotube formation as the cells advanced across the time-course (Fig. 2a). This was confirmed by increases in myoD and myogenin mRNA expression in both young and aged cells as differentiation progressed up to 72 h (Fig. 2b). However, myotube formation was less extensive in elderly cells, despite young and aged cells having identical numbers of starting myogenic cells. This age associated reduction in differentiation was confirmed with reduced myoD and myogenin gene expression at 72 h and 10 d (p ≤ 0.05), as well as by delayed increases in myogenin mRNA expression (Fig. 2b) in aged cells. A similar delay has been shown previously to be associated with impairing the fusion process and myotube formation in cells with an aged phenotype^32,33^. There were also differences in DNA methylation between aged and young cells during differentiation. Indeed, the interaction for a 2-way ANOVA (Age × Time) generated a list of 40,854 DMPs that were significantly altered with age and across the time-course of differentiation (FDR ≤ 0.05, Suppl. File 2a). With a more stringent FDR ≤ 0.01 and ≤ 0.001 there were still 9938 and 2020 DMPs respectively in aged cells versus young cells across all time points of differentiation (Suppl. File 2b and 2c respectively).

We next assessed changes in DNA methylation across time during differentiation (main effect for ‘time’) i.e. changes in DNA methylation overtime time that was visible in both young and old cells). However, we did not find time-related DMPs at our statistical cut off of FDR ≤ 0.05. This suggested DNA methylation was not considerably changing over the time course of HDMC differentiation itself. We therefore contrasted each timepoint of differentiation with the baseline (0 h timepoint) in young and aged cells to further examine this observation. Indeed, in young adult cells, there were only 1 and 14 DMPs at 72 h and 10 d respectively (FDR ≤ 0.05), and no DMPs at 7 d (FDR ≤ 0.05) compared to their baseline 0 h timepoint. In aged cells, there were no DMPs during early differentiation (72 h vs. 0 h, FDR ≤ 0.05) but 2,785 significant DMPs at 7 days of differentiation (7d vs. 0 h, FDR ≤ 0.05, Suppl. file 2d) and 404 DMPs at 10 days of differentiation (10 d vs. 0 h, FDR ≤ 0.05, Suppl. File 2e). Therefore, while the differentiation itself was not changing DMPs in young cells there were a significant number of DMPs at 7 days of differentiation in aged cells compared with their own 0 h timepoint. Using more stringent cut-offs (FDR ≤ 0.05 and change/difference in methylation greater than 2), at this 7-day time point in aged cells there were 1,229 DMPs at 7 d vs. 0 h (Suppl. File 2f.), with a balanced number of hypo and hypermethylated DMPs. Overall, this may suggest a more dysfunctional DNA methylation program during differentiation in aged human muscle cells compared with young cells. Conducting gene ontology analyses at this 7 day time point (Suppl. File 2g), it suggested that this altered methylation response in aged cells was enriched in GO terms: ‘regulation of localisation’, ‘regulation of cell communication’ and ‘regulation of signaling’ (Suppl. Figure 5; Suppl. File 2h,i,j respectively). DMPs within these ontologies also confirmed that there was a similar hypo and hypermethylation profile in aged cells at 7 days. Further, KEGG pathway analysis suggested the top enriched pathways at 7 d vs. 0 h in aged cells were: ‘axon guidance’, cholinergic synapse’, ‘adrenergic signaling in cardiomyocytes’ and ‘circadian entrainment’ (Suppl. File 2k). Finally, DMR analysis between young and aged cells at 7 days of differentiation identified two regions in 2 genes that had 5 or more CpG sites significantly differentially methylated (Suppl. File 2 l). The first being Chr3:155,394,096–155,394,303 (209 bp) located on gene PLCH1 that had 5 of its CpG’s hypomethylated. The second being a region non-annotated in location Chr2: 119,615,888–119,617,128 (1241 bp) that was hypermethylated on 5 CpG’s. Suggesting that enriched and differential methylation of these regions occurred at 7 d differentiation in aged cells that was not detected at 7 days in young adult cells.

### Aged muscle cells demonstrate hypermethylated signatures versus young muscle cells across differentiation, particularly at 7 days

We next wished to identify differences in DNA methylation in aged versus young HDMCs. The main effect for ‘age’ generated a significant differentially methylated (FDR ≤ 0.05) CpG list of 269,898 sites that were significantly changed in aged cells versus young cells. Even with a more stringent cut-off (FDR ≤ 0.01) there were still 159,235 sites significantly modified (Suppl. File 3a). Increasing the stringency to a difference of greater than 2 while keeping an FDR ≤ 0.01 identified the most highly significant 2719 DMPs with largest difference in methylation between aged and young cells (Suppl. File 3b). As with the aged versus young skeletal muscle tissue analysis, the majority of these DMPs in aged cells were hypermethylated (2017 out of 2719) versus hypomethylated (702 out of 2719) compared with young cells, visualised using hierarchical clustering (Fig. 2c). Two hundred and eleven out of these 2719 CpGs were located in islands and 97 were promoter associated. Gene ontology identified that aged cells demonstrated this significantly enriched hypermethylated profile in GO terms: ‘developmental process’, ‘anatomical structure development’, ‘anatomical structure morphogenesis’ (Suppl. File 3c) and also in KEGG pathways ‘axon guidance’, adherens junction’, ‘calcium signaling’, ‘focal adhesion’ and ‘protein digestion and absorption’ (Suppl. File 3d). DMR analysis (Suppl. File 3e) identified that a non-coding location on chr12:115,134,344–115,135,576 (1233 bp) that contained 13 CpG’s was hypermethylated in aged cells versus young cells. Further, there were 7 CpG’s that were hypermethylated in the region of the gene LY6G5C (Chr6:31,650,736–31,651,158, 423 bp). There was also region containing 8 CpGs of the HOXC10 gene just upstream of HOXC6 and MIR196 (Chr12:54,383,692–54,385,621, 1933 bp) that all demonstrated a hypomethylated profile in aged cells vs. young cells. Interestingly, on the same chromosome just upstream of the HOXC10 gene (Chr12:54,376,020–54,377,868, 1849 bp), within the lncRNA HOXC-AS3, there were another 6 CpGs that were hypomethylated in aged cells versus young cells. The concentration of hypomethylated CpGs in aged versus young cells in HOXC genes is interesting given the majority of CpG’s, more generally, were hypermethylated in aged cells versus young cells. With HOX genes being analysed in more detail below.

Given these changes, predominantly favouring hypermethylation (except the HOXC genes identified above) in aged cells versus young cells, we also performed contrasts between aged and young adult cells at each time point of differentiation. When comparing 0 h aged cells with young adult cells at 0 h we identified 738 DMPs between young and old cells (FDR ≤ 0.05, with a difference/change greater than 2), 79% of which were hypermethylated in aged versus young cells (Suppl. File 3f). Gene ontology analysis (Suppl. File 3g) revealed that the DMPs were in genes enriched for ‘cytoskeletal protein binding’ (Suppl. Figure; Suppl. File 3h), ‘developmental process’, ‘cell junction’, ‘cytoskeleton’ and ‘actin binding’ (Suppl. File 3i,j,k,l respectively). KEGG pathway analysis of 0 h aged versus 0 h young adult cells (Suppl. File 3 m) also suggested that there were significantly enriched hypermethylated pathways for ‘axon guidance’ (Suppl. Figure 6b; CpG List Suppl. File 3n), ‘insulin secretion’, ‘phospholipase D signaling’, ‘cAMP signaling pathway’ and ‘aldosterone synthesis and secretion’ (Suppl. Files 3o,p,q,r respectively). At 72 h d, there were 1,418 DMPs between aged and young adult cells (FDR ≤ 0.05), and with a difference/change greater than 2 there were 645 significant CpG sites between aged and young adult cells (Suppl. File 4a), 74% of which were hypermethylated in aged vs. young cells. Gene ontology analysis (Suppl. File 4b) revealed that the DMPs were amongst genes enriched for ‘cytoskeletal protein binding’ (Suppl. Figure 6c; Suppl. File 4c), ‘actin binding’ (Suppl. File 4d), ‘cytoskeleton’, ‘development process’, and ‘regulation of signaling’ (Suppl. Files 4e, f, g respectively). KEGG pathway analysis (Suppl. File 4h) showed enrichment for ‘Insulin secretion’ (Suppl. File 4i), ‘aldosterone synthesis and secretion’ (Suppl. File 4j) and ‘cAMP signaling pathway’ (Suppl. Figure 6d, Suppl. File 4k).

It was at 7 d of differentiation that we identified the largest number of DMPs between young and old cells (5524 DMPs at FDR ≤ 0.05, with a difference/change greater than 2, Suppl. File 5a). 74% of DMPs were hypermethylated in aged vs. young cells. Gene ontology analysis (Suppl. File 5b) revealed that DMPs were in genes enriched for ‘developmental process’ (Suppl. Figure 7a, Suppl. File. 5c), ‘anatomical structure morphogenesis’ (Suppl. File 5d), ‘neuron part’, ‘cytoskeletal protein binding’ and ‘cell junction’ (Suppl. File 5 e, f, g). KEGG analysis (Suppl. File 5 h) identified: ‘Focal adhesion’ (Fig. 2d; Suppl. file 5i), ‘adherens junction’ (Suppl. File 5j), ‘regulation of actin cytoskeleton’ (Suppl. File 5k), ‘cGMP-PKG signaling pathway’ Suppl. File 5l), ‘rap1 signaling pathway’ (Suppl. File 5m), as well as the muscle differentiation pathway of ‘PI3K-Akt signaling pathway’ (Suppl. Figure 7b, Suppl. File 5n). Among the DMRs at 7 d (Suppl. File 5o) was a region within HOXB1, containing 8 hypermethylated CpGs in aged vs. young cells. As in the above analysis, we also identified the same gene, LY6G5C, to be hypermethylated in 6 CpG sites in aged vs. young cells at 7 days, this time spanning a slightly larger region of 627 bp (Chr6:31,650,736–31,651,362) vs. 423 bp (Chr6:31,650,736–31,651,158) in the above analysis. At 10 d, we identified 288 DMPs (FDR ≤ 0.05 with a change greater than 2, Suppl. File 6a), 89% of which were hypermethylated in aged vs. young cells. Gene ontology analysis (Suppl. File 6b) revealed that the DMPs were in genes enriched for ‘GDP binding’ (Suppl. Figure 7c, Suppl. File 6c), ‘phosphotransferase activity’, ‘positive regulation of antigen receptor’, ‘mesoderm morphogenesis’ and ‘actin binding’ (Suppl. File 6d, e, f, g respectively). KEGG analysis (Suppl. File 6h) identified ‘regulation of actin cytoskeleton’ (Suppl. Figure 7d; Suppl. File 6i), ‘ErbB signaling pathway’ (Suppl. File 6j) and ‘selenocompound metabolism’ (Suppl. File 6k).

### Self-organising map (SOM) profiling of aged HDMCs confirms more frequent alterations in methylation in aged cells compared with young cells

**Figure 3:**
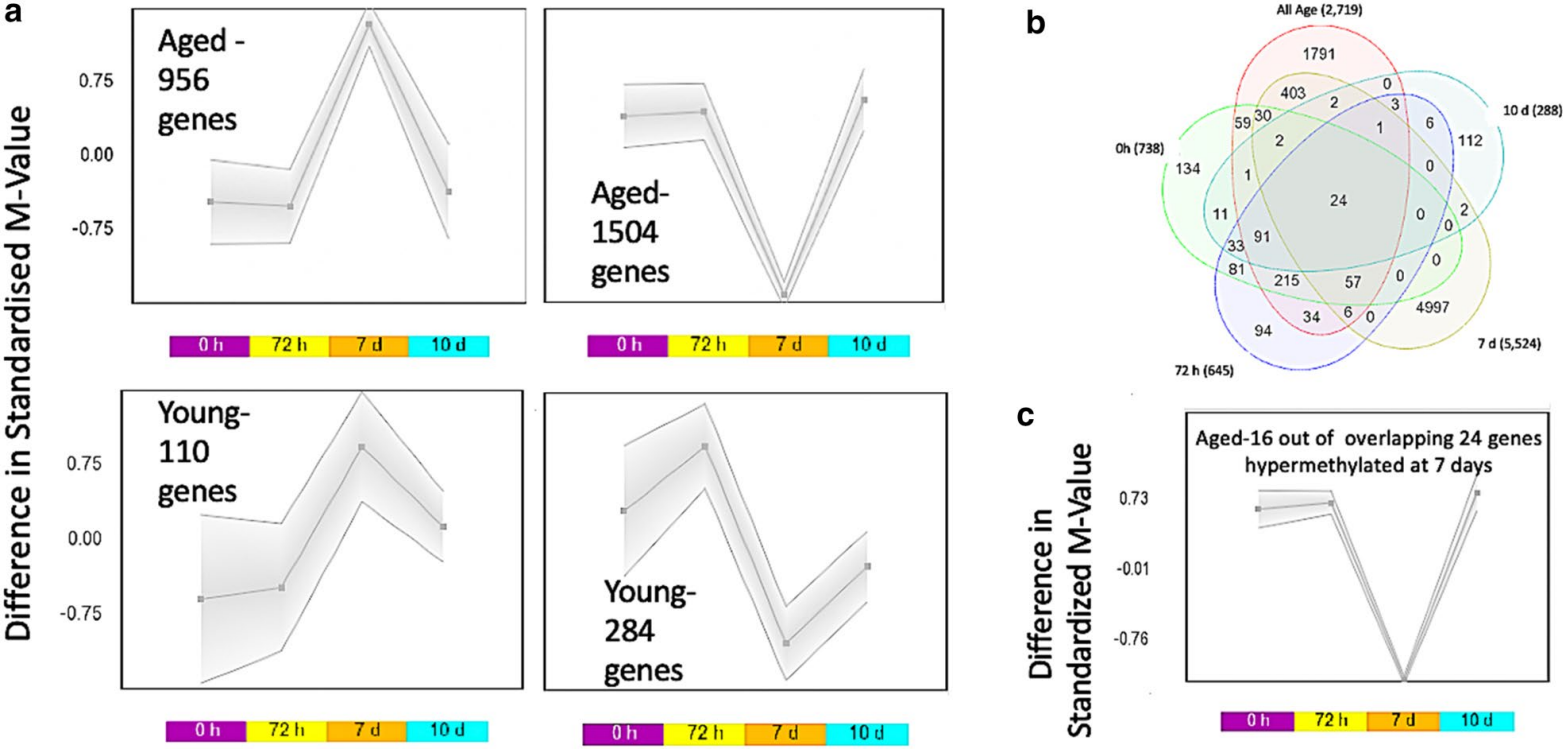
SOM profiling of DNA methylation over the time-course of differentiation in aged versus young adult HDMCs. (**a)** Demonstrates a larger number of hypomethylated and hypermethylated CpG sites in aged HDMCs, particularly at 7 days of differentiation, compared with young adult HDMCs. (**b)** Venn Diagram analysis depicting the 24 common CpG sites that were altered at every time point of differentiation between aged and young adult muscle HDMCs cells (0, 72 h, 7d and 10 d). **c**. As with the above analysis in 3a, SOM profiling identified that 16 out of these 24 CpG’s also demonstrated the most alterations in methylation at 7 days of differentiation in aged cells.

In order to further analyse temporal dynamics in methylation over the time course of differentiation in aged versus young cells, we conducted SOM profiling analysis of the 2719 DMPs generated above (Suppl. File 3b above). SOM analysis averages the methylation values for the group of samples within each condition to identify temporal profile changes in DMPs between aged and young cells over the time-course of differentiation. Aged cells demonstrated more significantly different DNA methylation changes earlier in the time-course of differentiation (between 72 h and 7 d) compared with young adult cells (Fig. 3a). When looking at aged cells temporal dynamics compared with young cells, out of the 2719 DMPs in aged cells 1,504 were hypomethylated and 956 hypermethylated at 7 days (confirming the aged cells ‘time’ main effect analysis above). With only a small number of genes demonstrating this altered profile at 7 days in young cells (284 hypomethylated and 110 hypermethylated out of the 2719 CpG list).

Subsequently, in order to identify DMPs that were significantly altered at every time point of differentiation *,* the significantly differentially methylated DMPs from young vs. aged cells across all time were overlapped (2719 CpG list), with the 0 h aged vs. 0 h young (738 CpG list), 72 h aged vs. young 72 h (645 CpG list), 7 d aged vs. 7 d young (5,524 CpG list), and the 10 d aged vs 10d young (288 CpG list) (Venn diagram, Fig. 3b). There were 24 genes that were identified across all HDMC analyses that were significantly differentially methylated (Suppl. File **7**a). They included 24 CpG’s on 16 genes: TSPAN9, RBM22, UBAP1, CAPZB, ZNF549, MBNL2, RMI1, CHRM5, RAB4A, C19orf21, MOBKL1A, ANAPC11, GAS7, PBX1, ELOVL2, FGGY. Furthermore, generating a SOM profile of temporal change in methylation over the time-course of differentiation in these 24 CpG’s, (Fig. 3c), also demonstrated that the majority of these DMPs in aged cells (16 out of 24 CpG’s) demonstrated altered methylation at 7 days (11 out of 16 CpGs on annotated genes: TSPAN9, RBM22, UBAP1, CHRM5, C19orf21, ELOVL2, MOBKL1A, ANAPC11, PBX1, CAPZB, FGGY; fully detailed in Suppl. File **7**a**)**.

### Distinguishing differentiation-specific CpG sites in aged cells versus those altered as a consequence of age alone

The data above suggest that aged cells demonstrate hypermethylation versus young adult cells, and that aged cells significantly altered their methylation profiles at 7 days of differentiation. Therefore, we conducted further analysis on the overlap of DMPs within aged cells at 7 d of differentiation (from the ‘time’ analysis above) and those that were changed as a consequence of age at 7 days (aged cells at 7 days versus young cells at 7 d). This enabled the identification of which methylation sites were altered, but also shared in both aged cell differentiation alone and as a consequence of age. Or, alternatively the sites that were simply changed with age and not differentiation process and vice versa. Indeed, overlapping the aged cells 0 h vs. 7 d (1229 DMP list) with the 7 d young cells significant 5524 DMP list, there were only 334 (206 hypermethylated, 128 hypomethylated) DMPs that were shared (Suppl. File. 8a). This suggested that differentiation itself modified only 334 DMPs (out of 1229) in aged cells that were also changed as a consequence of age (i.e. in aged vs. young cells at 7 days). The remaining 895 DMPs (1229 minus 334 DMPs; Suppl. File 8b) were differentiation specific to aged cells. Out of this 895 DMP list, an equal number were hypo and hypermethylated (458 hypo and 437 hypermethylated). Therefore, this overlap analysis also confirmed that data above, where over the time-course of aged cell differentiation itself the methylome is both hypo and hyper methylated on a similar number of DMPs, whereas aging alone predominantly hypermethylates (where out of 5524 DMPs at 7 d young vs. aged, 4061 were hypermethylated and only 1463 hypomethylated). This also suggested that the remaining 5190 (5524 minus 334 DMP list, equalling 5190 DMPs made up of 3910 hypermethylated vs. 1280 hypomethylated) were altered as a consequence of aging alone (Suppl. File 8c).

### Hypermethylation for a small number of CpG sites is similarly altered in aged muscle cells in-vitro from the in-vivo tissue niche

**Figure 4:**
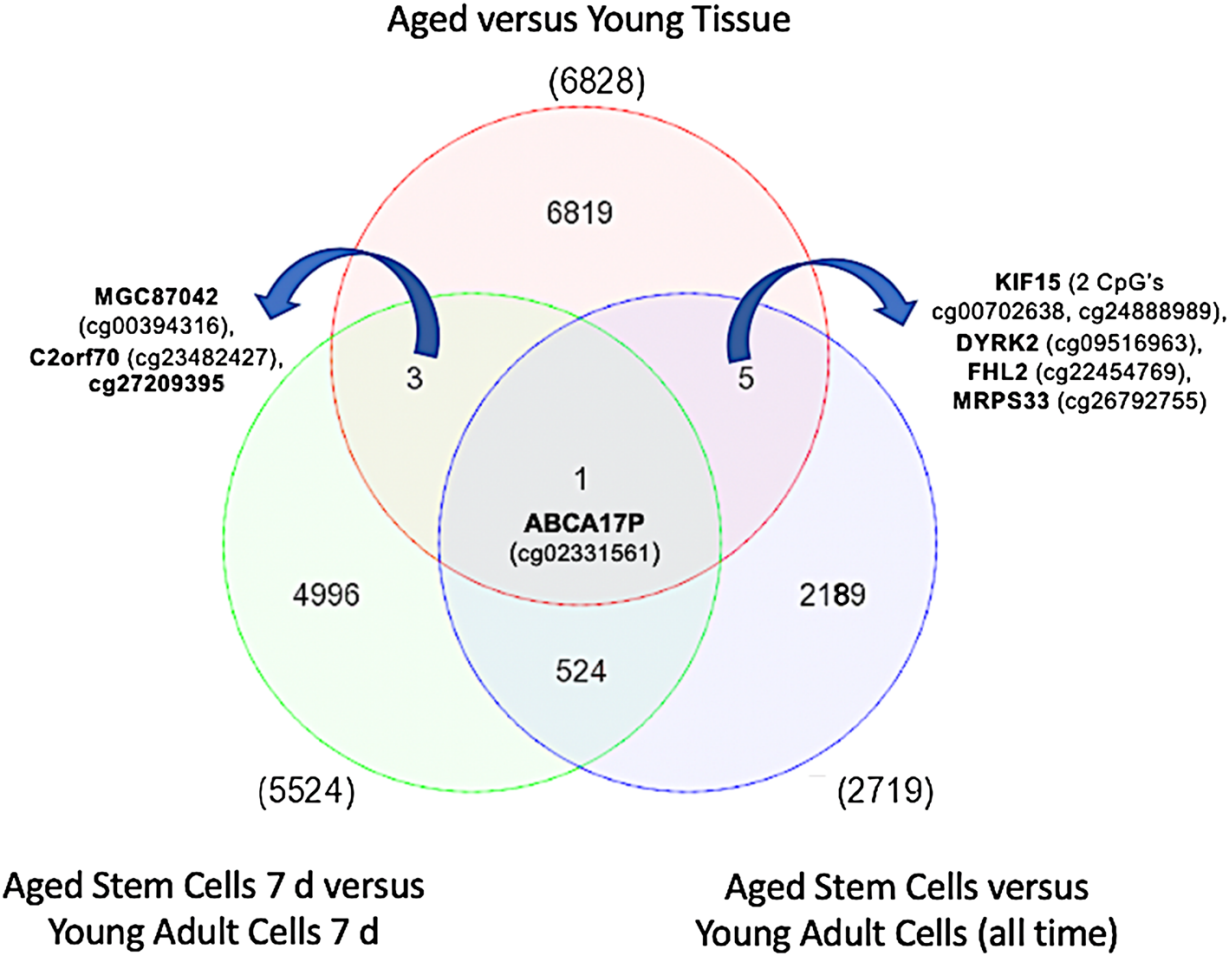
Venn diagram of common overlapping significant DMPs between aged skeletal muscle tissue and HDMCs. Overlap of the tissue and aged cells identified 6 common DMPs on genes KIF15 (2 CpG’s Cg00702638 & cg24888989), DYRK2 (cg09516963), FHL2 (cg22454769), MRPS33 (cg26792755) and ABCA17P (cg02331561). Given that aged cells demonstrated the majority of changes in methylation at 7 days versus young cells. When overlapping the 7 d most significantly differentially methylated CpG lists, 4 DMPs (out of the 6 DMPs identified above) were also identified, including: MGC87042 (cg00394316), C2orf70 (cg23482427), ABCA17P (cg02331561) and cg27209395 (not an annotated gene). Once more, all of these DMPs (with the exception of C2orf70, cg23482427) were hypermethylated in the tissue analysis as well as the HDMCs. With ABCA17P (cg02331561) highlighted across all DMP lists.

Tissue and cell PCA plots demonstrated that methylation of even late differentiated cells was vastly different to tissue, suggesting that HDMCs had extensively different methylation profiles to tissue (Suppl. Figure 8). However, in order to compare if there were any similarly altered DMPs in aged HDMCs that were also altered in aged tissue, we overlapped DMP lists from the tissue and HDMC analysis described above. Six CpG’s that were identified in the 6828 DMP list from the aged versus young tissue analysis, as well as highlighted in the 2719 DMP list in the HDMC analysis (Fig. 4). These included: KIF15 (2 CpG’s Cg00702638 & cg24888989), DYRK2 (cg09516963), FHL2 (cg22454769), MRPS33 (cg26792755), ABCA17P (cg02331561). All of these genes were hypermethylated in the tissue analysis as well as the cells. Given that aged cells demonstrated differential methylation at 7 days versus young cells. When comparing 7 d aged vs. 7 d young cell list (5,524 DMP list), 4 DMPs (out of the 6 DMPs identified above) were also identified in the 6,828 tissue DMP list, including: MGC87042 (cg00394316), C2orf70 (cg23482427), ABCA17P (cg02331561) and cg27209395 (not an annotated gene). Once more, all of these DMPs (with the exception of C2orf70, cg23482427) were hypermethylated in the tissue analysis as well as the HDMCs. With ABCA17P (cg02331561) highlighted across all DMP lists (Fig. 4).

### Altered methylation in the HOX family of genes in aging skeletal muscle tissue and HDMCs

**Figure 5:**
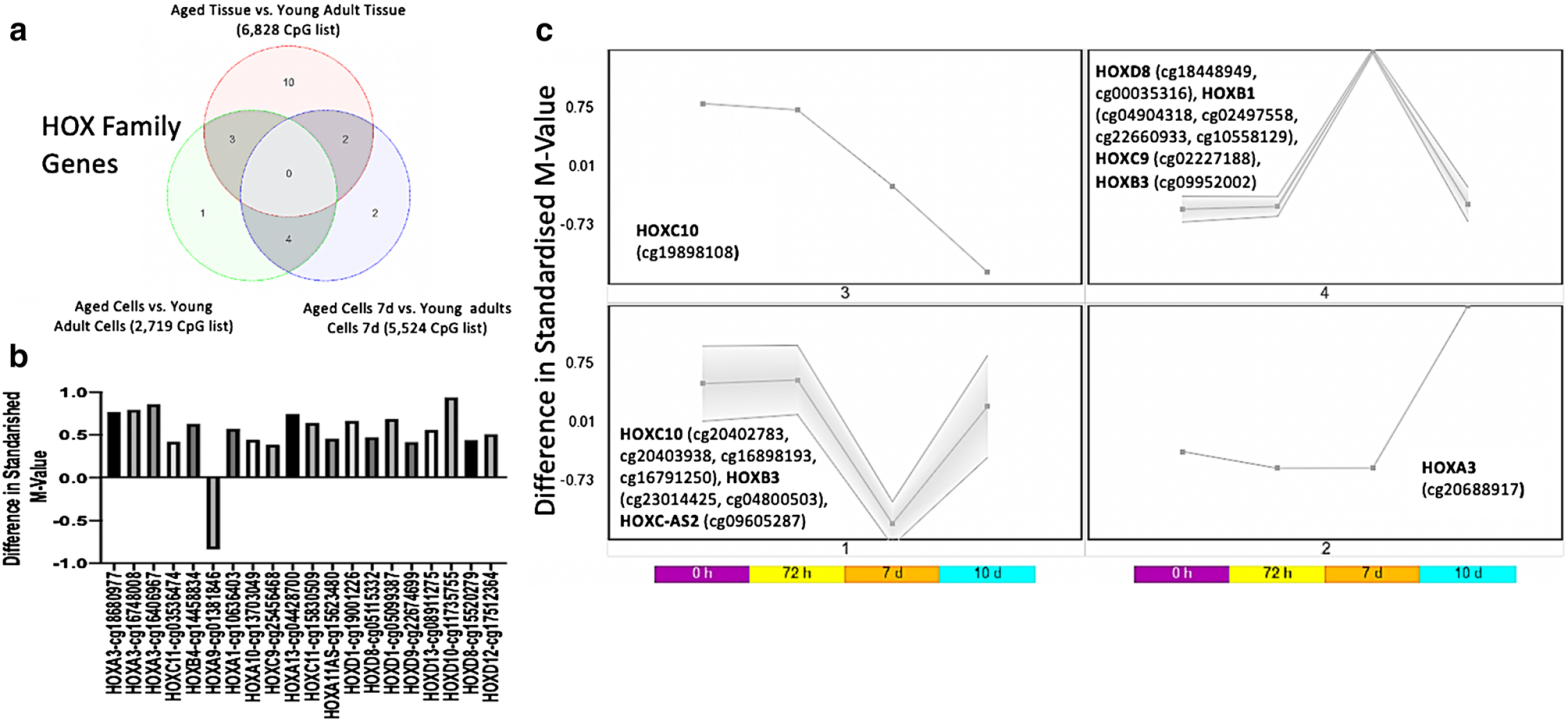
HOX family of genes and their DNA methylation in aged tissue and HDMCs. (**a**) Venn diagram analysis identifying 9 overlapping differentially methylated HOX genes: including HOXD10, HOXD9, HOXD8, HOXA3, HOXC9, HOXB1, HOXB3, HOXC-AS2 and HOXC10 (note this Venn diagram analysis is by ‘gene symbol’ not ‘probe cg’ (CpG site), as some HOX genes also had more than 1 CpG per gene symbol, full CpG lists are located in Suppl. Figure 9a,b,c,d). (**b)** All HOX family genes by CpG site (cg probe) differentially methylated in aged compared with young skeletal muscle tissue, predominantly all demonstrating hypermethylation. (**c)** SOM profiling depicting the temporal regulation of DNA methylation in aged HDMCs as they differentiate in CpGs located amongst the HOX family of genes (depicting the 9 HOX genes altered, by gene symbol, in both the tissue and cells). The majority of these HOX CpG sites were differentially methylated at 7 days of differentiation in the aged cells.

In the above analyses in aged versus young cells (all time points) there were 8 CpG’s with altered methylation within the region of the HOXC10 gene just upstream of HOXC6 and MIR196 (Chr12:54,383,692–54,385,621, 1933 bp). Also, on the same chromosome just upstream of the HOXC10 gene (Chr12:54,376,020–54,377,868, 1849 bp) within lnRNA HOXC-AS3 there were another 6 CpG’s that were altered in aged cells versus young cells. Similarly, within the 5524 DMP list at 7 d in aged cells versus 7 d young adult cells (Suppl. File 5o), the time point most affected by age in the cell analysis, we also identified that a region of the HOXB1, located in Chr17:46,607,104–46,608,568 that contained 8 CpG’s that were hypermethylated within a 1465 bp region. Therefore, we further analysed all the HOX gene changes in the significantly differentially methylated aged vs. young tissue (6,828 DMP list) and identified that CpG’s within HOXD10, HOXD9, HOXD8, HOXA3, HOXC9, HOXB1, HOXB3, HOXC-AS2 and HOXC10 were significantly differentially methylated across all analyses, including the aged vs. young tissue (6,828 DMP list; Suppl. File 9a), aged versus young adult cells (all time) 2,719 DMP list (Suppl. File 9b) as well as the 7 d aged versus 7 d young cell analysis 5,524 DMP list (Suppl. File 9c). A Venn diagram analysis depicted the overlap in common gene symbols for these three gene lists (Fig. 5a, Suppl. File 9d). It was also demonstrated that the majority of these HOX genes were hypermethylated in aged tissue (Fig. 5b; Suppl. File. 9a). In the cell analysis across all time, these HOX genes also displayed altered methylation at 7 days of differentiation in aged cells versus young cells, therefore confirming the temporal profile in methylation described above at 7 d was also the case for these HOX genes. Finally, when SOM-profiling these 9 HOX genes by symbol (17 CpGs as some HOX genes contained more than one CpG site), over the time-course of differentiation based on the main effect for ‘age’ 2,719 significantly differentially methylated CpG list. Eight CpG sites across HOX family genes: HOXD8 (cg18448949, cg00035316), HOXB1 (cg04904318, cg02497558, cg22660933, cg10558129), HOXC9 (cg02227188) and HOXB3 (cg09952002) were hypermethylated at 7 days, whereas and 7 CpGs across HOXC10 (cg20402783, cg20403938, cg16898193, cg16791250), HOXB3 (cg23014425, cg04800503), HOXC-AS2 (cg09605287) were hypomethylated at 7 days (Fig. 5c; Suppl. File 9e). This meant distinct HOX genes were hypermethylated (HOXD8, HOXC9, HOXB1) and hypomethylated (HOXC10, HOXC-AS2) at 7 days in aged cells versus young adult cells, except for one of these genes, HOXB3, that contained 1 CpG that was hypermethylated versus 2 CpGs that were hypomethylated.

**Figure 6:**
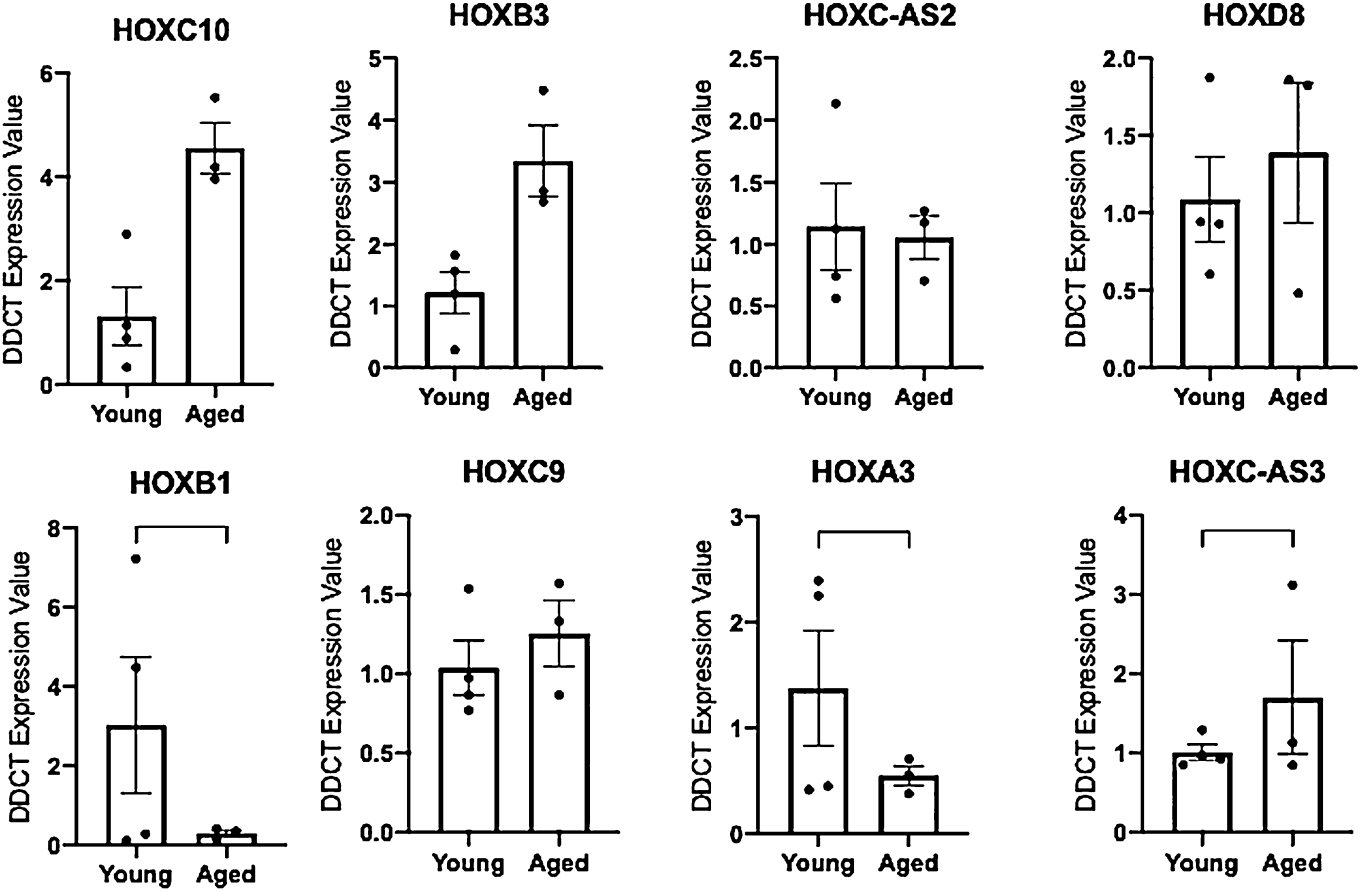
Gene expression of the HOX family of genes in aged compared to young HDMCs at 7 days of differentiation**.**

We next analysed the gene expression of the HOX genes that changed at the methylation level in both the tissue and cell analysis (by gene symbol- HOXD8, HOXA3, HOXC9, HOXB1, HOXB3, HOXC-AS2 and HOXC10). Interestingly, in aged tissue, despite displaying hypermethylation in all these HOX genes versus young tissue, the genes were not suppressed at the gene expression level, which may have been expected, yet all elevated (Suppl. Figure 9). However, in the aged cells when analysing these genes at the expression level at 7 days of differentiation (including HOXD8, HOXA3, HOXC9, HOXB1, HOXB3, HOXC-AS2 and HOXC10, as well as HOXC-AS3 identified in the above in the cell analysis only) (Fig. 6), we identified that there was significantly reduced gene expression in gene HOXB1 (Fig. 6), that was inversely related with increased HOXB1 methylation. Where in the 5524 DMP list at 7 d in aged cells versus 7 d young adult cells (Suppl. File 5o), we previously identified that a region of the HOXB1 located in Chr17:46,607,104–46,608,568, contained 8 CpG’s that were hypermethylated, as well as HOXB1 Cg’s: cg04904318, cg02497558, cg22660933, cg10558129 being identified as hypermethylated in the 2719 significant main effect for ‘age’ DMP list above (Suppl. File 3b). There was also significantly increased gene expression for HOXC-AS3, with this gene identified earlier in the analysis as having reduced (hypo)methylation. Indeed, hypomethylation occurred in 6 CpG’s in a region upstream of the HOXC10 gene (Chr12:54,376,020–54,377,868, 1849 bp) within the HOXC-AS3 gene. Interestingly, HOXC10 also demonstrated an average increase in gene expression, however, it was not statistically significant (Fig. 6). Finally, HOXA3 also demonstrated an inverse relationship between gene expression and methylation, with significantly reduced expression (Fig. 6) and corresponding hypermethylation (at 10 not 7 days of differentiation) in aged cells (see Fig. 5c). Overall, HOXB1, HOXC-AS3 and HOXA3 demonstrated an inverse relationship with CpG methylation and gene expression in aged versus young cells.

### Effect of physical activity on methylation status of HOX family genes

Given that aging generally hypermethylated the genome, we tested the hypothesis that increasing physical activity may oppositely regulate DNA methylation and may be associated with increasing hypomethylation of these HOX genes. We thus performed multiple regression analysis using methylation data and the level of physical activity of 30 endurance-trained men. As in the above analysis, we also found that CpG-sites associated with physical activity (P < 0.05) were significantly enriched with HOX genes (137 of 1219 CpG-sites, Fisher’s exact test OR = 1.7, P = 2.3*10–8, Suppl. File 10a**)**. Where we determined that highly active men had hypomethylated HOXB1 (cg10558129, P = 5.2*10^–4^), HOXA3 (cg16406967, P = 0.03), HOXD12 (cg17512364, P = 0.008) and HOXC4 gene (cg13826247, P = 0.014) compared to less active men on the same sites identified above in the aging data. Furthermore, we identified hypomethylation of HOXA3 on several additional sites (cg21134232, P = 0.0014, cg25768734, P = 0.0002; cg27539480, P = 0.039; cg00431187, P = 0.026; cg03483713, P = 0.045; cg15982700, P = 0.026; cg23806243, P = 0.013). Full methylome analysis of the physical activity dataset can be found in Suppl. File 10b, with details of all HOX sites in Suppl. File 10a). Given that we identified the opposite trend in aged muscle and cells, particularly HOXB1 and HOXA3 that were hypermethylated in aged tissue and HDMCs, and with reduced gene expression in aged cells. These findings suggest that increasing levels of physical activity are associated with increasing reductions in methylation (hypomethylation) in these HOX genes compared to age-related changes that are associated with increasing methylation (hypermethylation).

## Discussion

In the present study we first aimed to investigate the methylome in aged skeletal muscle tissue and differentiating HDMCs compared with young adults, in order to identify important epigenetically regulated genes in both aging skeletal muscle tissue and muscle derived cells. As with previous studies^18,27^, and by using more recent, higher coverage array technology, we identified that aged skeletal muscle tissue demonstrated a considerably hypermethylated profile compared with young adult tissue. We also demonstrated that these hypermethylated profiles in aged tissue were enriched in gene ontology pathways including, ‘regulation of muscle system process’ and KEGG pathways ‘pathways in cancer’, a pathway that incorporates previously well described molecular pathways in the regulation of skeletal muscle mass such as; focal adhesion, MAPK signaling, PI3K-Akt-mTOR signaling, p53 signaling, Jak-STAT signaling, TGF-beta and Notch signaling, as well as the other significantly enriched pathways of ‘rap1 signaling’, ‘axon guidance’, and ‘hippo signaling’. This was also the first study to profile DNA methylation over the entire time-course of skeletal muscle differentiation (0, 72 h, 7 and 10 d) using the highest coverage 850 K methylation assays. In primary muscle-derived cell cultures, isolated from aged and young adults with the same proportion of myogenic cells, we identified that aged HDMCs also demonstrated hypermethylated profiles versus young adult cells. This hypermethylation was enriched in: ‘axon guidance’, ‘adherens junction’ and ‘calcium signaling’ pathways. Furthermore, we identified that the process of cellular differentiation itself did not significantly affect DNA methylation in young cells, however aged cells demonstrated differential methylation profiles particularly at 7 d of differentiation in GO terms: ‘regulation of localisation’, ‘regulation of cell communication’, and ‘regulation of signaling’. Also, in the majority of significantly differentially methylated CpG sites that were altered during the process of differentiation, aged cells demonstrated significantly hypermethylated profiles when compared with young adult cells. Again, this was particularly evident at 7 d of differentiation including CpG’s located within: ‘focal adhesion’, ‘adherens junction’ and ‘regulation of actin cytoskeleton’ pathways, as well as the well-known muscle cell differentiation pathway, ‘PI3K-AKT signalling’. This corresponded with reductions in myoD and myogenin, and delayed increases in myogenin gene expression in aged compared with young adult cells.

We were also able to identify that a small number of CpG sites hypermethylated in aged tissue were also hypermethylated in aged cells, with CpG’s located on genes: KIF15, DYRK2, FHL2, MRPS33, ABCA17P. This suggested, that perhaps these CpG’s retained their methylation status in-vitro after being isolated from the in-vivo niche. However, it is worth noting that this was a very small number of CpG’s compared with the thousands of CpG sites significantly differentially methylated in the aged tissue versus young adult tissue, and those also observed to be significantly different in the aged versus young muscle cells. This was suggestive that the majority of the hypermethylated CpG’s observed in aged tissue in-vivo were generally not retained on the same CpG sites in the isolated aged HDMCs in-vitro. Also, PCA plots of muscle tissue versus HDMCs demonstrated extensively different profiles, suggesting that isolated cells, even late differentiated muscle cells, are quite different epigenetic representations of contrasting cellular entities versus muscle tissue. This also perhaps indicates that hypermethylation of DNA within myonuclei (the predominant source of DNA in the tissue samples) maybe therefore be unique to that observed in the muscle derived cells that contains expansions of other muscle derived cell populations. Indeed, retention of methylation during aging has been previously observed in artificially aged muscle cells^19^. In skeletal muscle tissue, retention of DNA methylation has been observed after skeletal muscle growth, even after a subsequent period of muscle loss, suggesting an epigenetic memory at the DNA methylation level^21,22,49^. However, the relative contribution of methylation from myonuclei or satellite cells (or other resident cell types in muscle tissue) to this epigenetic memory effect, and how long these retained profiles can last (e.g. past 22 weeks in the Seaborne et al., 2018 study) has not been determined. However, interestingly based on the present studies data, we could hypothesise that myonuclear hypermethylation in the tissue with age is quite different to the hypermethylation observed in HDMCs as they expand and differentiate. Perhaps, suggesting that environmental stimuli and aging could affect methylation profiles in the myonuclei differently than those in muscle progenitor including, for example, satellite cells. A hypothesis that requires further investigation, perhaps using single-cell DNA/RNA analysis of the different cell population proportions resident in cells derived from skeletal muscle tissue biopsies compared with cultured HDMCs. Finally, it is important to mention that the human derived muscle cells were comprised of both muscle and non-muscle cells in an attempt to capture the changes across the skeletal muscle ‘milieu’ of cell types. Therefore, as alluded to above, methylation profiles reflect both the differentiation of muscle cells but also expansion of predominantly fibrogenic and a minority of other adherent muscle derived cells. As suggested above with respect to specific methylation changes in myonuclei versus satellite cells, it will be important in future studies to undertake single-cell DNA/RNA analysis to identify the specific contribution of different cell types to the methylation profiles observed in aged HDMCs. Indeed, exciting recent work suggests different methylation profiles from myonuclear versus interstitial cells in response to a hypertrophic stimulus in skeletal muscle^62^.

Importantly, in both tissue and HDMC analysis we also identified that the homeobox (HOX) family of genes were significantly enriched in differentially methylated region analysis, showing several (e.g. 6–8) CpGs to be methylated within chromosomal regions on these genes in aged compared with young adults. In particular we identified HOXC10 (just upstream of HOXC6) and HOXB1 as having several CpGs differentially methylated. Therefore, closer analysis of all HOX gene associated CpG changes across both tissue and cell differentiation data identified that CpG’s located within: HOXD10, HOXD9, HOXD8, HOXA3, HOXC9, HOXB1, HOXB3, HOXC-AS2 and HOXC10 were all significantly differentially methylated across these analyses. In aged tissue the majority of these HOX genes were hypermethylated. In the cell analysis, these HOX genes displayed the most significantly altered methylation at 7 days of differentiation in aged versus young cells. Furthermore, distinct HOX genes were hypermethylated (HOXD8, HOXC9, HOXB1) and hypomethylated (HOXC10, HOXC-AS2) at 7 days in aged cells versus young adult cells. Gene expression analysis also demonstrated an inverse relationship with DNA methylation. Where hypermethylation of HOXB1 and HOXA3 was associated with reduced gene expression of the same genes, and hypomethylation of HOXC-AS3 was associated with this genes increased expression in aged versus young cells.

HOX genes are evolutionary conserved members of the homeobox superfamily, with 39 HOX genes found in the mammalian genome. They are ‘homeotic’ genes coding for transcription factors, with a fundamental role in the determination of cellular identity. They were first shown to be important in embryogenesis in drosophila melanogaster (fruit fly)^63^. HOX genes have also been reported to alter with age in non-muscle tissues^64,65^. In muscle they have been described to morphologically identify the hindlimb tissues during development^66–69^, but have also been demonstrated to be activated in satellite cells^70–72^, and as markers of hindlimb derived myoblasts^70^. In particular HOXC10, demonstrated 8 CpG’s, just upstream of HOXC6 and miR-196; Chr12:54,383,692–54,385,621, 1933 bp that all demonstrated a hypomethylated signature in aged versus young HDMCs. Where miR-196 has previously been demonstrated to regulate HOX gene expression in adipose tissue^73^. There were also 4 CpG sites hypomethylated, particularly at 7 days of differentiation in aged versus young cells. Indeed, HOXC10 has been identified to determine hindlimb identity^66,68^. Together with HOXC10 hypomethylation, we also demonstrated average (yet not significant) increases in HOXC10 gene expression at 7 days in aged cells versus young cells. Counterintuitively to our data, previously HOXC10 upregulation has been associated with satellite cell activation in skeletal muscle in response to Roux-en-Y gastric bypass (RYGB) surgery^74^, as well as being a marker for hindlimb specific satellite cells. Interestingly, there was lower expression of HOXC10 observed in exercised rats^75^, which is perhaps more intuitive with the data provided here, where aged cells demonstrated an increase. However, HOXC10 requires more experimentation to define its mechanistic role in aging skeletal muscle with HOXC10 with physical exercise being discussed below. Interestingly, the hypomethylation of the HOXC10 (Chr12:54,383,692–54,385,621, 1933 bp) occurred in 8 CpG’s on the same chromosome just upstream of the HOXC10 gene (Chr12:54,376,020–54,377,868, 1849 bp) within the lncRNA HOXC-AS3, where there were another 6 CpG’s that were hypomethylated in aged cells versus young cells (See Suppl. Figure 10 for visualisation of HOXC10 and HOXC-AS3, their methylation and genomic location). HOXC-AS3 is a long coding RNA (lcRNA) with currently no known data in skeletal muscle, with some literature in cancer and mesenchymal stem cell (MSC) fields^76^. Indeed, MSC’s administered with silencing HOXC-AS3 prevented bone loss by enhancing HOXC10 expression ^77^, and HOXC-AS3 upregulation has also been linked with aggressive cancer^78^. Interestingly, we were able to identify that together with associated hypomethylation, in a region close to the lcRNA HOXC-AS3, there was significantly increased gene expression of HOXC-AS3 (and an average, yet not significant increase in HOXC10 gene expression) in aged muscle cells at 7 days of differentiation. Given the data above in bone and cancer, HOXC-AS3 upregulation appears to be pro-growth and linked with expression of HOXC10, therefore their increase in the current study maybe hypothesised to be a co-operative and compensatory drive to maintain aged muscle. However, this hypothesis is speculative, and more work needs to be conducted as to the role of HOXC10 and HOXC-AS3 and their potential cooperative mechanisms of action in aged skeletal muscle.

We also identified that HOXB1 was hypermethylated with increased gene expression in aged cells at 7 days. HOXB1 has been demonstrated to be hypermethylated in inflamed muscle of children with Juvenile Dermatomyositis (JDM)^79^. This is interesting given aged skeletal muscle is known to be chronically inflamed^80,81^, where we also demonstrate this hypermethylated profile in aged cells. HOXA3 was hypermethylated with reduced gene expression in aged cells. However, there is currently little to no work on this gene in skeletal muscle^69^ and therefore this gene requires future investigation. It is however worth noting that gene expression in the aged tissue in particular was not expected given the methylation data. Where aged tissue demonstrated an increase in gene expression of HOX genes with the majority demonstrating hypermethylation. This may be due one of the elderly patients’ donors demonstrating much greater expression versus all the other donors for some of the HOX genes. Therefore, in order to confirm this HOX gene expression would require confirmation in a larger population of aged patients. Other limitations to the study include a mixed cohort of male and female aged individuals compared to the young adult group being all male. However, PCA analysis of male and female aged samples did not show differences between methylation profiles and we also removed sex (X and Y) chromosome probes from the analysis. Furthermore, the muscle tissue and cells were isolated from two sites, the gluteus medius and vastus lateralis in the aged samples compared with only the vastus lateralis in the young adult samples. This is important to note as the gluteus medius and vastus lateralis have different fibre type proportions. Therefore, the data should be viewed with that caveat in mind. Despite this, the changes in methylation of the sites observed were identified across samples from both biopsy sites, suggesting that these changes occurred in aged individuals across muscle types.

**Figure 7:**
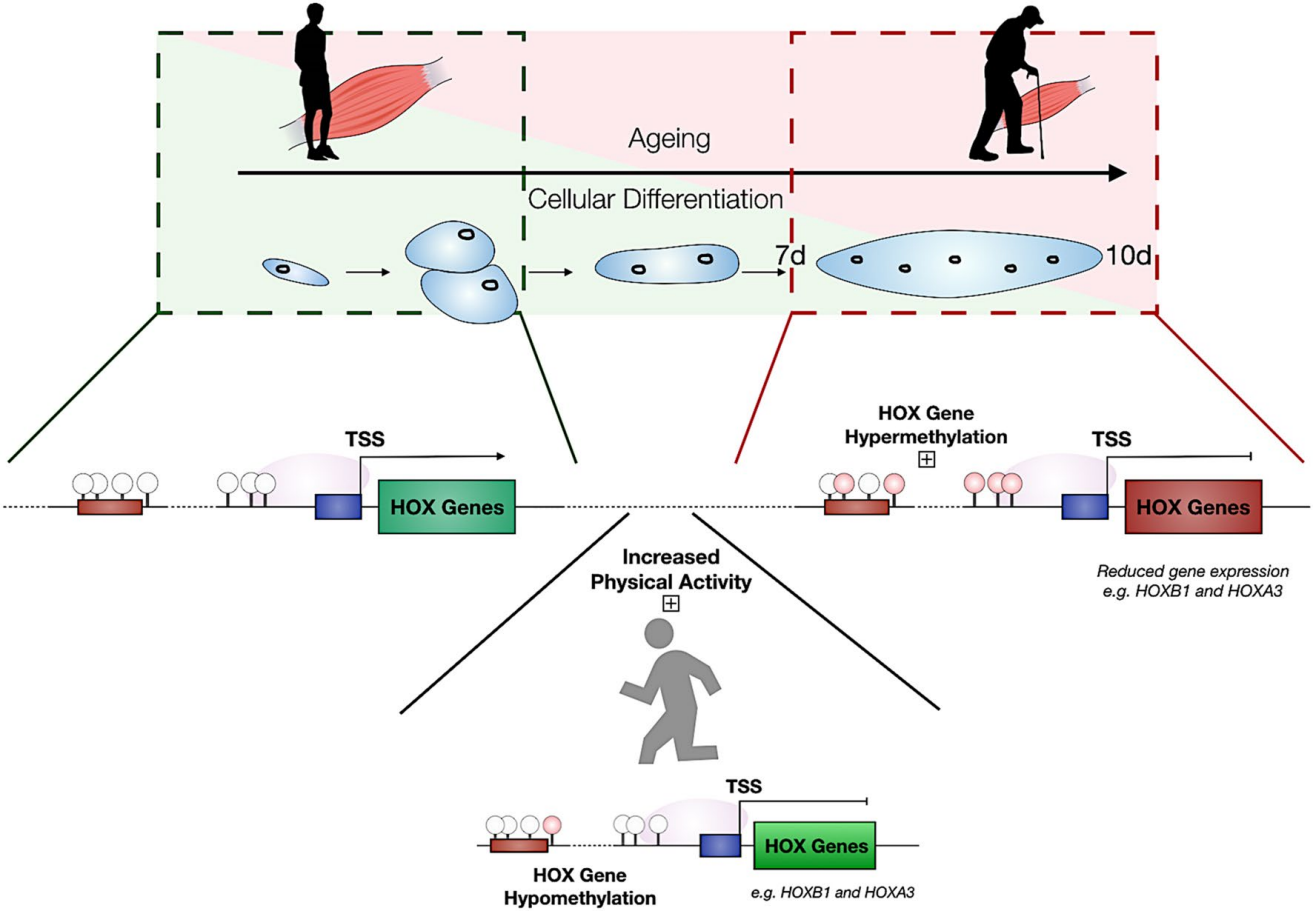
Schematic of the overarching main results. Hypermethylation of the genome and the HOX genes in aging skeletal muscle tissue (RED box) compared with young adult tissue (GREEN BOX). Dysregulation of HOX genes in aged cells particularly at 7 days of differentiation in muscle derived cells. Inverse methylation (hypermethylation- red circles) with gene expression (reduced- red rectangle) in aging cells particularly for HOXB1 and HOXA3. Increased physical activity levels are associated with the hypomethylation (fewer red circles more white circles) of the same two genes, HOXB1 and HOXA3.

Finally, with aging evoking a hypermethylated signature in tissue and aged HDMCs, it was also interesting to speculate that physical exercise, that has been shown to hypomethylate the genome^20–22^, could therefore be ‘anti-ageing’ at the epigenetic level. Indeed, this hypothesis was supported indirectly in the present study and by previous literature, where the aged tissue analysis in the present study identified the top significantly enriched KEGG pathway as, ‘pathways in cancer’. A pathway that incorporates well known pathways important in skeletal muscle mass regulation, including: Focal adhesion, MAPK, PI3K-Akt, mTOR, p53 signaling, Jak-STAT, TGF-beta and Notch signaling. Where, this pathway was also the top enriched hypomethylated pathway after acute and chronic resistance exercise^22^. While, perhaps the significance of these larger pathways such as ‘pathways in cancer’ can be inflated in methylation analysis^82^, and therefore should be viewed with some caution. This data is still intriguing and perhaps suggests that exercise could perhaps reverse the hypermethylated profiles in these pathways in aged muscle. Therefore, in the present study, given that we identified the HOX family of genes to be extensively differentially methylated in aged tissue and HDMCs, we went on to determine that increasing physical activity levels (endurance exercise) in healthy young adults was associated with larger reductions in HOXB1 and HOXA3 methylation (hypomethylation). This was opposite to the changes we observed with age in both muscle tissue and HDMCs, that demonstrated hypermethylation with age. This also provided evidence to suggest that increased physical activity could perhaps reverse the age-related epigenetic changes in the HOX genes. Importantly, HOXA3 has also been shown to be hypomethylated on multiple sites after resistance exercise training with retention of hypomethylation for site cg12434681 during detraining and retraining^21^. Suggesting, that this HOX gene is also hypomethylated with exercise training and possesses an epigenetic memory from earlier exercise training as previously described by our group^21^. Unpublished work by our group also suggests that acute sprint exercise in human muscle can hypomethylated the same HOXA3 CpG site (cg00431187) that was oppositely hypermethylated in aging and also demonstrated hypomethylation in individuals that have higher physical activity levels. However, more research into the effect of exercise in an aged population and the changes in HOX methylation status will be required in the future to confirm these findings. Our overarching main results from this study are summarised schematically in Fig. 7.

## Conclusion

Overall, for the first time we demonstrate that altered methylation of a large number of the HOX genes are epigenetically differentially regulated in aged human skeletal muscle tissue and during differentiation in aged HDMCs. In particular HOXB1, HOXA3 and HOXC-AS3 (and to a certain extent, HOXC10) also demonstrated significantly inversed changes in gene expression in aged cells. Finally, that increased physical activity may help prevent the age-related epigenetic changes observed in these HOX genes.

## Supplementary information

Suppl. Figure 1

Suppl. Figure 2

Suppl. Figure 3

Suppl. Figure 4

Suppl. Figure 5

Suppl. Figure 6

Suppl. Figure 7

Suppl. Figure 8

Suppl. Figure 9

Suppl. Figure 10

Suppl. File 1

Suppl. File 2

Suppl. File 3

Suppl. File 4

Suppl. File 5

Suppl. File 6

Suppl. File 7

Suppl. File 8

Suppl. File 9

Suppl. File 10
