# Supplementary material for: DNA methylation across the genome in aged human skeletal muscle tissue and muscle-derived cells: the role of HOX genes and physical activity
Source: Sci Rep. 2020 Sep 21;10:15360. doi: 10.1038/s41598-020-72730-z (PMC7506549; doi:10.1038/s41598-020-72730-z)
Supplement: Supplementary file 4 — Suppl. Figure 4 [file 41598_2020_72730_MOESM4_ESM.pdf]

Title: DNA methylation across the genome in aged human skeletal muscle tissue and stem cells: The role of HOX genes and physical activity

Authors: Turner DC, Gorski PP, Maasar MF, Seaborne RA, Baumert P, Brown AD, Kitchen MO, Erskine RM, Dos-Remedios I, Voisin S, Eynon N, Sultanov RI, Borisov OV, Larin AK, Semenova EA, Popov DV, Generozov EV, Stewart CE, Drust B, Owens DJ, Ahmetov II, Sharples AP.

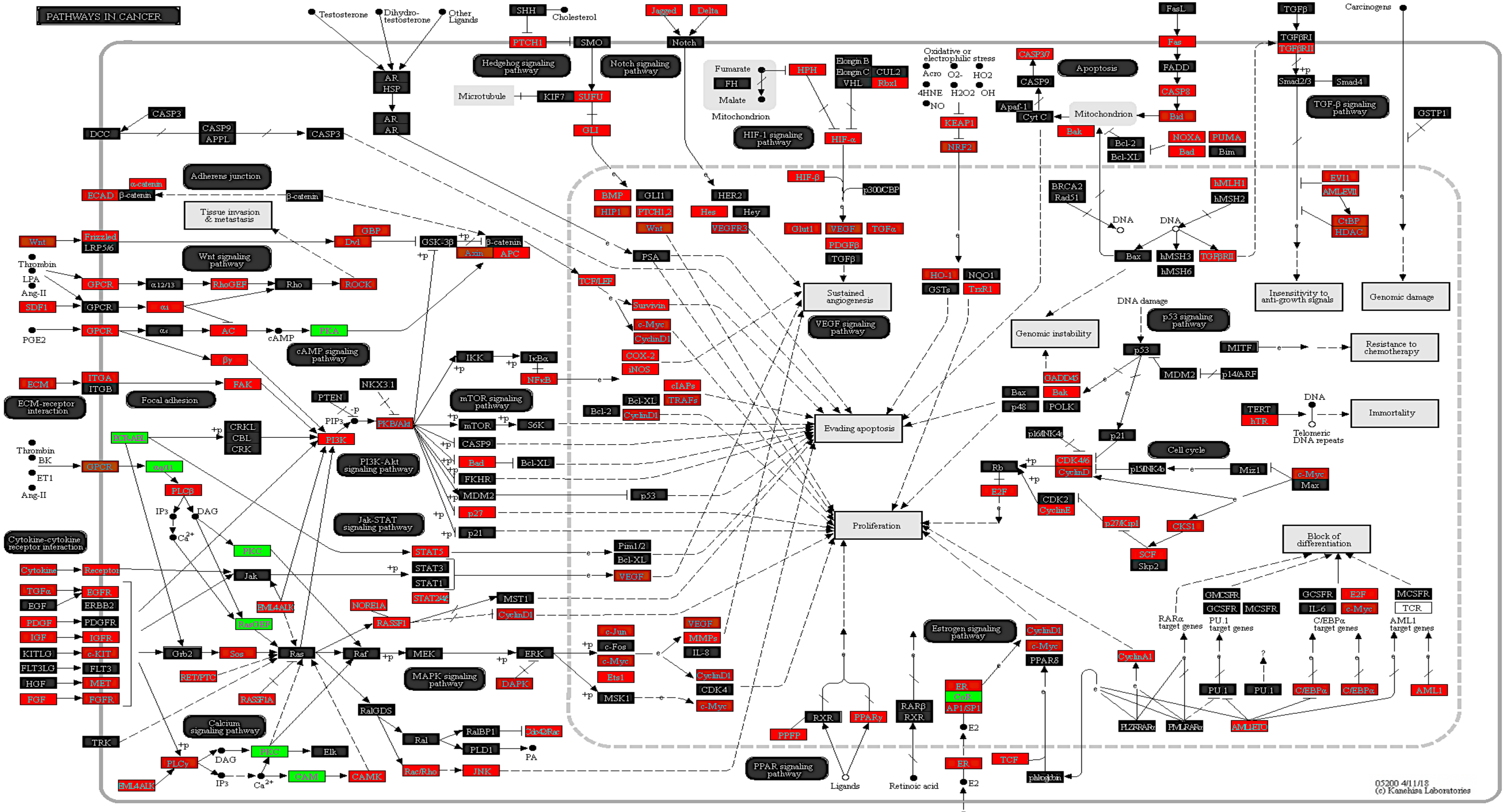

Suppl. Figure 4. **The top significantly enriched KEGG pathway 'Pathways in Cancer' in aged compared with young adult human skeletal muscle tissue**. This pathway includes well known pathways in skeletal muscle regulation such as: Focal adhesion, MAPK signaling, PI3K-Akt-mTOR signaling, p53 signalling, Jak-STAT signaling, TGF-beta and Notch signalling. In this pathway 266 out of 277 CpG's were hypermethylated (RED) and only the remaining 11 hypomethylated (GREEN) in aged compared young adult muscle tissue. Note, because some genes have multiple CpGs per gene, Partek Pathway (Partek Genomics Suite) used to create this figure, selects the CpG with the lowest p-value (most significant) and uses the beta-value for that particular CpG to colour the pathway diagram. This is accurate therefore in situations where CpG's on the same gene have the same methylation pattern (e.g. all hyper or hypomethylated). However, where multiple CpG's on the same gene have a different methylation pattern (e.g. some hypomethylated and others hypermethylated), only the most significant methylated CpG is chosen and represented in the image. However, in this instance the majority of CpG's (even when there was more than one CpG per gene) were hypermethylated, so in this case the figure depicts an accurate representation of the data. Full CpG lists including the sites that were hypo and hypermethylated in this pathway are included in Suppl. File 1h.

Title: DNA methylation across the genome in aged human skeletal muscle tissue and stem cells: The role of HOX genes and physical activity

Authors: Turner DC, Gorski PP, Maasar MF, Seaborne RA, Baumert P, Brown AD, Kitchen MO, Erskine RM, Dos-Remedios I, Voisin S, Eynon N, Sultanov RI, Borisov OV, Larin AK, Semenova EA, Popov DV, Generozov EV, Stewart CE, Drust B, Owens DJ, Ahmetov II, Sharples AP.
