# Supplementary material for: DNA methylation across the genome in aged human skeletal muscle tissue and muscle-derived cells: the role of HOX genes and physical activity
Source: Sci Rep. 2020 Sep 21;10:15360. doi: 10.1038/s41598-020-72730-z (PMC7506549; doi:10.1038/s41598-020-72730-z)
Supplement: Supplementary file 8 — Suppl. Figure 8 [file 41598_2020_72730_MOESM8_ESM.pdf]

8

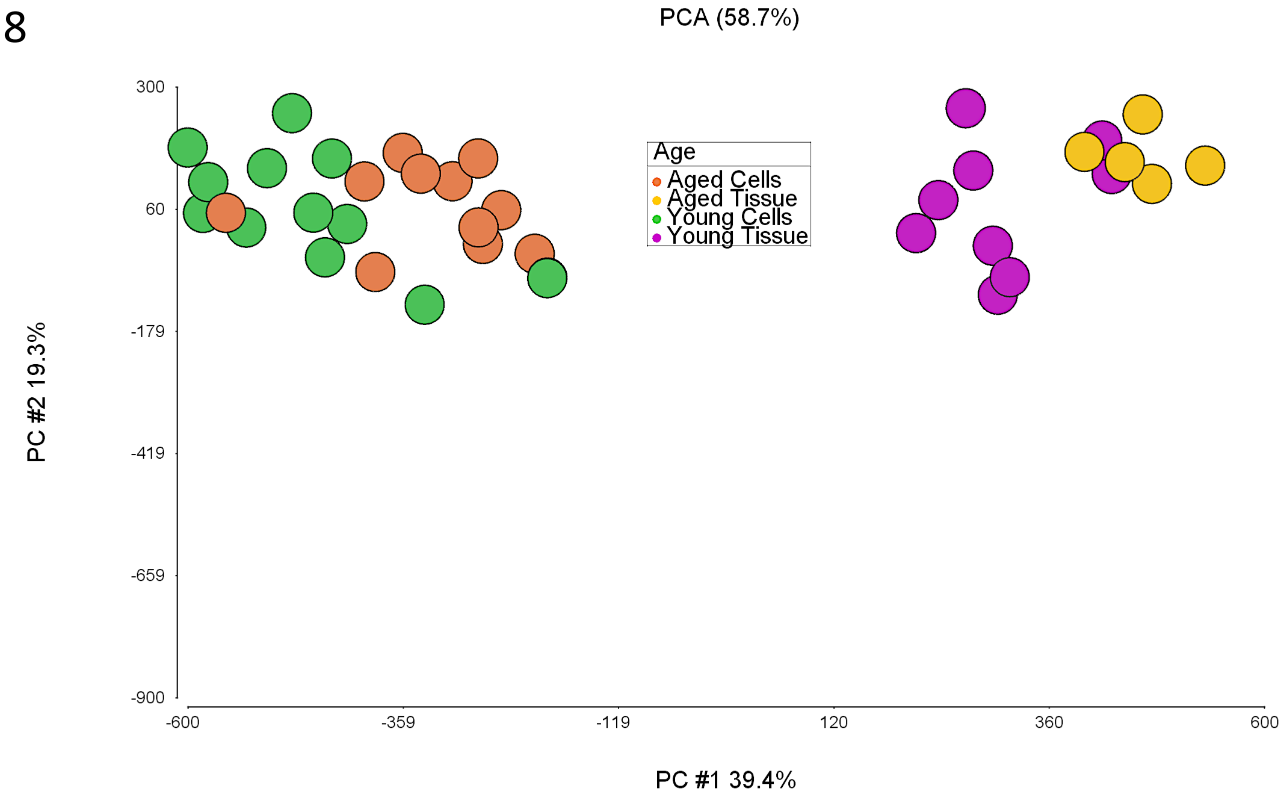

Suppl. Figure 8. PCA of aged and young adult skeletal muscle tissue versus aged and young adult isolated muscle stem cells across 0, 72 hr, 7 and 10 d of differentiation. Demonstrating methylation profiles of isolated muscle stem cells, even at late stages of differentiation, are vastly different compared with skeletal muscle tissue methylation profiles.

Title: DNA methylation across the genome in aged human skeletal muscle tissue and stem cells: The role of HOX genes and physical activity

Authors: Turner DC, Gorski PP, Maasar MF, Seaborne RA, Baumert P, Brown AD, Kitchen MO, Erskine RM, Dos-Remedios I, Voisin S, Eynon N, Sultanov RI, Borisov OV, Larin AK, Semenova EA, Popov DV, Generozov EV, Stewart CE, Drust B, Owens DJ, Ahmetov II, Sharples AP.
